# *Corchorus olitorius* exhibits antiproliferative potential supported by metabolic profiling and integrative biological analyses

**DOI:** 10.1038/s41598-025-02717-1

**Published:** 2025-05-25

**Authors:** Salma Sameh, Maha R. A. Abdollah, Ahmed M. Elissawy, Eman Al-Sayed, Rola M. Labib, Lan Ye, Fang-Rong Chang, Abdel Nasser B. Singab

**Affiliations:** 1 Department of Pharmacognosy, Faculty of Pharmacy, Ain-Shams University, Cairo, 11566 Egypt; 2 Department of Pharmacology, Faculty of Pharmacy, The British University in Egypt, El Sherouk City, Egypt; 3 Center of Drug Discovery Research & Development, Faculty of Pharmacy, Ain-Shams University, Cairo, 11566 Egypt; 4 Cancer Centre, the Second Hospital of Shandong University, 247 Beiyuan Street, Jinan, 250033 Shandong China; 5 School of Pharmacy and Graduate Institute of Natural Products, College of Pharmacy, Kaohsiung Medical University, Kaohsiung, 80708 Taiwan

**Keywords:** Herbal nutraceuticals, *Corchorus*, Malvaceae, Antiproliferative, Anti-angiogenic, UPLC-ESI-MS/MS, Drug discovery, Chemistry

## Abstract

Herbal nutraceuticals could be employed as alternative or complementary routes for alleviating cancer. *Corchorus olitorius* (Malvaceae) was employed traditionally in the management of tumors. The study aimed to investigate the antiproliferative activity of *C. olitorius* leaves. *In vitro* cytotoxic and anti-angiogenic activities of *C. olitorius* were estimated. The bioactive fraction was subjected to *in vivo* study on BALB/ c female mice using Ehrlich Ascites Carcinoma model. UPLC-ESI-MS/MS analysis was done to determine the phytometabolites followed by *in silico* studies on the major identified compounds. The bioactive fraction possessed potent *in vitro* activity against A549 cells with IC_50_ = 7.8 µg/mL and exhibited strong anti-angiogenic activity. The *in vivo* study revealed the safety of the fraction and confirmed its anticancer activity. The tumor volume in the fraction treated group was reduced by 33.7% compared to the control group. UPLC-ESI-MS/MS analysis led to the identification of 25 compounds belonging to different chemical classes. The *in silico* pharmacodynamic profile revealed that the compounds exhibited agreeable binding affinities toward EGFR, CDK2 and VEGF-A comparable to the standard drugs. *C. olitorius* is a promising herbal nutraceutical from which effective chemopreventive and anticancer formulations can be developed following further in depth studies.

## Introduction

Cancer is considered to be a reason for large numbers of death worldwide. It affects the patients’ lives on physical and emotional levels. The currently used chemotherapy and radiotherapy lead to serious adverse effects. In addition, they impose serious economic burdens on healthcare systems and patients^1^. Current studies showed that phytochemicals could be employed as alternative or complementary routes for alleviating cancer by re-establishing normal epigenetic marks that are changed due to tumorogenesis. Herbal nutraceuticals are considered to be dietary supplements exhibiting potent health benefits and could be employed in the prevention and treatment of cancer, as bioactive phytochemicals are able to decrease the growth and proliferation of tumor cells^2^. Chemoprevention is an important practical approach able to suppress the carcinogenesis process by different mechanisms^3^. Chemopreventive agents exert their effects by inducing apoptosis by activating caspases. Apoptosis is considered the most effective form of defense against cancer^4^. Moreover, angiogenesis is responsible for malignant cells development and metastasis. Thus, cancer prevention and treatment can be achieved by targeting tumor angiogenesis. Plants are considered to be rich in polyphenolic compounds with antioxidant activity which can target cancer and inhibit angiogenesis^5,6^.

Medicinal plants are invaluable sources of bioactive compounds, primarily due to their diverse secondary metabolites^7^, which contribute to their therapeutic properties^8^. LC-MS/MS-based metabolomics approach is the most comprehensive analytical tool possessing high speed and sensitivity, which accelerates the identification of the metabolites of interest before performing the isolation step. This technique is considered highly powerful for drug discovery from plant-derived natural products^9–12^.

*Corchorus olitorius*, belonging to family Malvaceae and commonly known as wild okra, nalta jute or mulukhiyah, is an annual herbaceous plant that grows up to 90–120 cm height^13,14^. The plant is a highly nutritious green leafy vegetable rich in vitamins and minerals and has been widely employed in traditional and ayurvedic medicine for the management of gonorrhoea, ascites, pain, piles, anemia, fever, inflammation, and tumors, and for relieving engorged blood vessels^15–18^. The different organs of plants are rich in various interesting classes of phytochemicals such as cardiac glycosides, carotenoids, phenolics, fatty acids, polysaccharides, triterpenes, steroids, minerals, proteins, and vitamins^13,19–21^. The plant exhibited various pharmacological activities, such as antioxidant, anti-inflammatory, hepatoprotective, anti-obesity, antimicrobial, antiviral, antidiabetic, analgesic, immunostimulant, cardioprotective, and antitumor^13,22,23^. Previous reports have revealed that the plant elicited promising* in vitro* cytotoxic activity against metastatic melanoma (CaCi 1962 and LiGh 1927B), human melanoma (SK-MEL28), and breast cancer (MCF-7) cell lines^24–26^.

*C. olitorius* is considered a superfood and an important herbal nutraceutical widely consumed in Egypt in the form of viscous green soup. The use of this plant dates back to the time of Pharaohs, and the plant is a symbol of Egyptian homeland. Egypt’s ancient history revealed that the plant was exclusively employed by Egyptian pharaohs, royal families and the nobility class owing to its high nutritional value^27^. Therefore, it was interesting to explore the potential *in vitro* and *in vivo* anticancer activity as well as the *in vitro* anti-angiogenic effect of *C. olitorius* leaves growing in Egypt for the first time, to determine the phytoconstituents responsible for the activities, and to evaluate the in silico pharmacokinetic and pharmacodynamic profiles of the bioactive compounds in order to verify their traditional use in tumor management.

## Results

### *In vitro* assessment of the cytotoxic activity of the 70% ethanol extract and fractions of *C*. *olitorius*

The cytotoxic activity of the 70% ethanol extract, *n*-hexane, ethyl acetate fractions, and aqueous residue were tested at a concentration of 20 µg/mL against A549 (adenocarcinoma human alveolar basal epithelial cells), HepG2 (human liver cancer cell line) and MDA-MB-231 (invasive ductal carcinoma) cell lines. The results revealed that the ethyl acetate fraction was the most active among the tested samples with a percentage cell inhibition of 81.2, 12.3, and 44.9% against the tested cell lines, respectively. Moreover, the ethyl acetate fraction exhibited an IC_50_ = 7.8 µg/mL against A549 cells showing its potent activity. The results of the cytotoxic study are listed in the Supplementary Materials Table S1.

### Anti-angiogenic activity of the 70% ethanol extract and fractions of *C. olitorius*

The anti-angiogenic activity of the 70% ethanol extract, *n*-hexane, ethyl acetate fractions, and aqueous residue on endothelial progenitor cells were tested at a concentration of 50 µg/mL. The results revealed that the ethyl acetate fraction exhibited a very strong anti-angiogenic activity with percentage cell survival less than 0 at the tested concentration. The results of the anti-angiogenic activity were displayed in the Supplementary Materials Table S2.

### *In vivo* assessment of the anti-proliferative effect of the ethyl acetate fraction of *C. olitorius* on Ehrlich ascites carcinoma

**Fig. 1:**
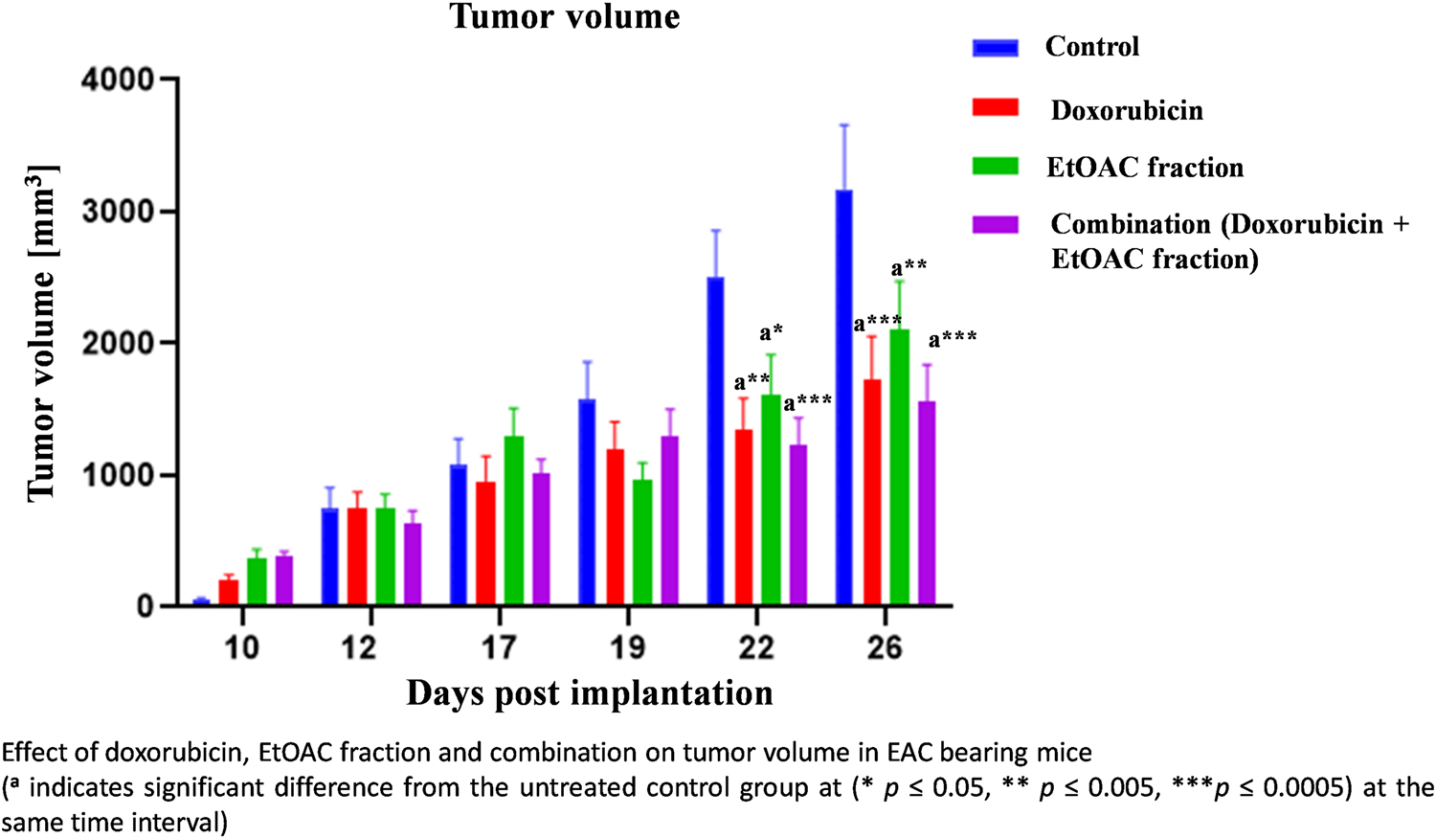
Effect of doxorubicin, EtOAC fraction and combination on tumor volume in EAC bearing mice.

Mice were divided into 4 groups; regarding the tumor volume, the bar chart (Fig. 1) represents the recorded tumor volume of the 4 groups measured on days 10, 12, 17, 19, 22 and 26 post implantation of the Ehrlich Ascites Carcinoma cells. On day twelve post implantation, it was obvious that the tumor volumes of all groups increased with no significant differences between groups showing the success of the model. The average tumor volume in mice thighs was equal to 723 mm^3^ prior treatment. Treatment started on day 12 post implantation. On the day 22 and 26, it was observed that all the treated groups exhibited significant decrease in tumor volume compared to the control group. It is important to note that on day 26 post implantation, doxorubicin reduced tumor volume by 45.8% compared to the control group, while, EtOAC fraction treated group and the combination treated group exhibited 33.7 and 50.5% reduction in tumor volume, respectively compared to the control group. Moreover, no significant difference was observed between the treated groups which proves the anticancer activity of the ethyl acetate fraction of *C. olitorius* leaves.

**Fig. 2:**
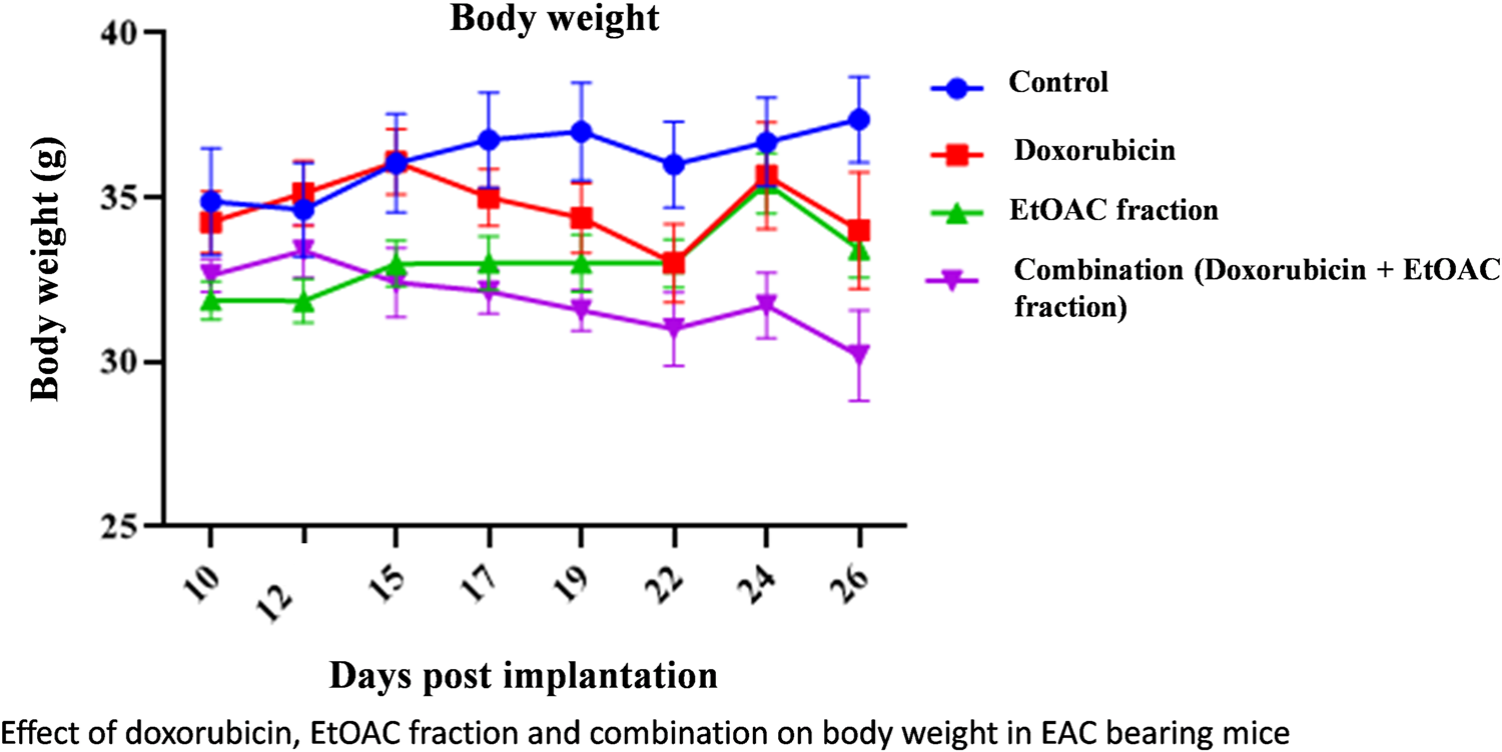
Effect of doxorubicin, EtOAC fraction and combination on body weight in EAC bearing mice.

Regarding the body weight (Fig. 2), a significant difference was observed between the combination and the control group on days 17, 19, 22, 24 and 26 post implantation. No significant difference was observed between the ethyl acetate fraction treated group and the untreated control group throughout the experiment. Thus, the findings prove the safety of the ethyl acetate fraction of *C. olitorius* leaves as anticancer agent.

Moreover, it was observed that two mice from group II treated with doxorubicin died on the day 24 post implantation, while, in group IV treated with the combination, one mouse died on the day 17 post implantation, one mouse died on the day 24 post implantation and one mouse died on the day 26 post implantation. These findings proved the toxicity of doxorubicin. No mortality was recorded in the ethyl acetate fraction treated group which support its safety. Furthermore, the results revealed that the combination treated group elicited higher mortality rate than the doxorubicin treated group. Thus, a synergistic cytotoxic action was observed in the combination treated group. Therefore, it is suggested to reduce the doxorubicin dose in the combination treated group.

### Histopathological study on tumor tissues and heart

**Fig. 3:**
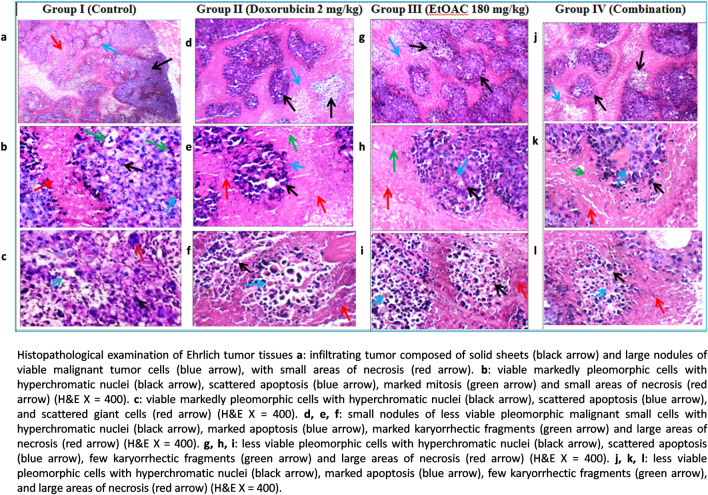
Histopathological examination of Ehrlich tumor tissues.

By examining the tumor tissues of the untreated control (group I), we observed the presence of infiltrating tumors composed of sheets and large nodules of viable markedly pleomorphic cells with hyperchromatic nuclei. Moreover, the tumor cells exhibited scattered apoptosis and few karyorrhectic fragments. The tissues also exhibited marked mitosis, scattered giant cells, and small areas of necrosis, as shown in Fig. 3. Concerning the doxorubicin-treated group (group II) receiving 2 mg/kg bw doxorubicin I.P, three times a week, the results showed infiltrating tumors composed of small nodules of less viable markedly pleomorphic cells with hyperchromatic nuclei, marked apoptosis, marked karyorrhectic fragments, and large areas of necrosis as shown in Fig. 3. Moreover, the ethyl acetate fraction-treated group (group III) received 180 mg/kg bw of *C. olitorius* ethyl acetate fraction I.P, three times a week, expressed infiltrating tumor composed of large nodules of less viable markedly pleomorphic cells with hyperchromatic nuclei, scattered apoptosis, few karyorrhectic fragments, and large areas of necrosis, as shown in Fig. 3. Furthermore, group IV receiving 2 mg/kg bw doxorubicin plus 180 mg/kg bw *C. olitorius* ethyl acetate fraction I.P, three times a week, showed an infiltrating tumor composed of small nodules of less viable markedly pleomorphic cells with hyperchromatic nuclei, marked apoptosis, few karyorrhectic fragments, and large areas of necrosis, as shown in Fig. 3. Results are displayed in the Supplementary Materials in Table S3.

**Fig. 4:**
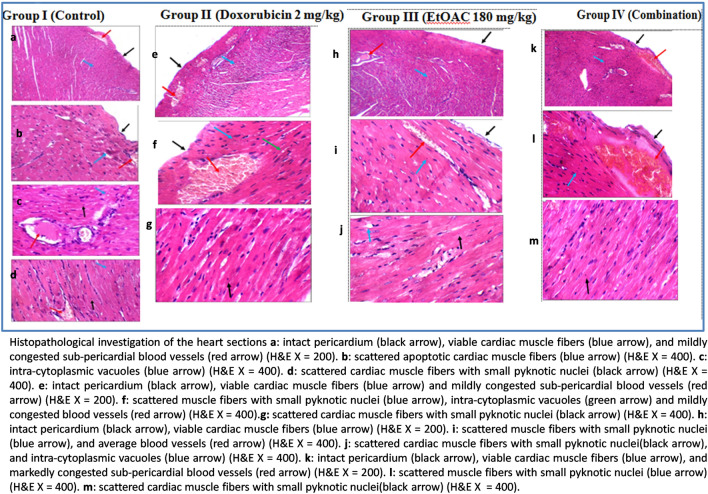
Histopathological investigation of the heart sections.

Concerning the histopathological examination of the heart sections, all the groups expressed an intact pericardium. The untreated control group I and II (doxorubicin 2 mg/kg bw) had mildly congested blood vessels, whereas group III (EtOAC 180 mg/kg bw) had average blood vessels. Moreover, group IV (combination treatment) exhibited markedly congested blood vessels. The results revealed that treatment with the ethyl acetate fraction exerted a protective effect on the heart. It is recommended to reduce the dose of doxorubicin in the combination treatment group due to the potent cytotoxic effect of the ethyl acetate fraction to avoid adverse reactions. The results are shown in Fig. 4.

### Immunohistochemical study

**Fig. 5:**
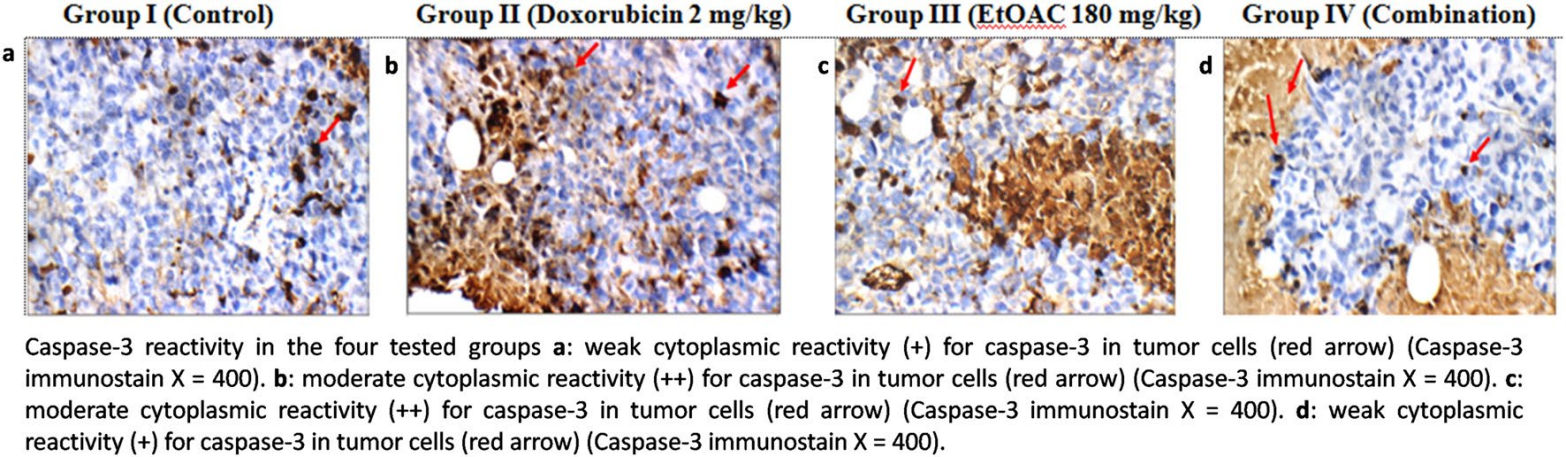
Caspase-3 reactivity in the four tested groups.

**Fig. 6:**
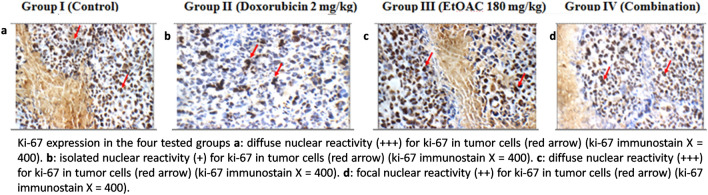
Ki-67 expression in the four tested groups.

The results of the immunohistochemical study concerning caspase-3 and Ki-67 showed that group I elicited weak cytoplasmic reactivity for caspase-3 and diffuse nuclear reactivity for Ki-67 in tumor cells. Moreover, group II exhibited moderate cytoplasmic reactivity for caspase-3 and showed isolated nuclear reactivity (+) for Ki-67, whereas group III exhibited moderate cytoplasmic reactivity for caspase-3 and diffuse nuclear reactivity for Ki-67 in tumor cells. In addition, group IV exhibited weak cytoplasmic reactivity for caspase-3 in tumor cells and focal nuclear reactivity for Ki-67. The results are listed in Supplementary Materials Table S4 and illustrated in Figs. 5 and 6.

### Determination of total phenolic and total flavonoid contents

The total phenolic contents were determined in the ethyl acetate fraction of *C. olitorius* leaves using Folin-Ciocalteu reagent. The result was expressed as gallic acid equivalent (GAE) per milligram of the ethyl acetate fraction and was equal to 37.36 ± 2.43 µg GAE/mg. Also, the aluminum chloride method was utilized to assess the total flavonoid contents. The result was shown as rutin equivalent (RE) per milligram of the ethyl acetate fraction and was equal to 11.82 ± 0.27 µg RE/mg.

### UPLC-ESI-MS/MS analysis

UPLC-ESI-MS/MS analysis led to tentative identification of 25 compounds belonging to various chemical classes such as benzimidazoles, flavonoids, acetophenones, phenolic acids, triterpenes, steroids, chlorophyll catabolites, secoiridoids, fatty acids, and fatty amides displayed in the Supplementary Materials Fig. S1. The compounds were identified by comparing their mass and MS/MS spectra with the previously reported literature on *Corchorus* species and online database (Mass Bank). The tentatively identified compounds were listed in Table 1 along with their chromatographic and MS/MS data. It is important to note that the major compounds belong to flavonoids, fatty acids and their derivatives and fatty amides. Notably, four of the identified compounds, namely, quercetin glucoside (2), dicaffeoyl quinic acid (4), and corchorifatty acid F (trihydroxy octadecadienoic acid) (6) were previously identified in *C. olitorius* leaves^28,29^. In addition, trihydroxy octadecenoic acid (7), octadecadienoic acid ethyl ester (9), oleamide (22) were previously identified in family Malvaceae^30–32^.

**Table 1:** Tentatively identified compounds in EtOAC soluble fraction of *C. olitorius* leaves.

Peak No	R_t_ (min.)	Compounds	Mol. Formula	[M-H]^-^	[M + H]^+^	MS^2^ ion fragments (*m/z*)	Class	References
1	1.64	Carbendazim	C_9_H_9_N_3_O_2_	–	192	160, 132	Benzimidazole	^33,34^
2	5.62	Quercetin glucoside	C_21_H_20_O_12_	463	465	304, 303, 184, 123, 300	Flavonoid glycoside	^35,36^
3	5.93	Dimethoxy hydroxyacetophenone	C_10_H_12_O_4_	–	197 219 [M + Na]	179, 137, 107	Acetophenones	^38^
4	6.53	Dicaffeoyl quinic acid	C_25_H_24_O_12_	515	539 [M + Na]	353	Phenolic acid	^39^
5	7.92	Ethyl caffeate	C_11_H_12_O_4_	207	209	179, 161, 135	Phenolic acid	^40,41^
6	8.69	Corchorifatty acid F (trihydroxy octadecadienoic acid)	C_18_H_32_O_5_	327	351 [M + Na]	291, 229, 211, 183, 171	Fatty acid	^29,48,49^
7	9.28	Trihydroxy octadecenoic acid	C_18_H_34_O_5_	329	353 [M + Na]	229, 211, 171	Fatty acid	^49,50^
8	9.48	Ferulic acid pentosyl	C_15_H_18_O_8_	325	–	193	Phenolic acid	^42,43^
9	10.46	Octadecadienoic acid ethyl ester	C_20_H_36_O_2_	307	–	235, 185, 125, 71	Fatty acid	^51^
10	10.61	Zahnic acid	C_30_H_46_O_7_	517	–	455, 437, 429	Triterpene	^45^
11	11.52	Hydroxy eicosapentaenoic acid	C_20_H_30_O_3_	–	319	274, 230, 85, 57	Fatty acid	^52^
12	11.62	Hydroxy eicosapentaenoic acid isomer	C_20_H_30_O_3_	–	319	274, 230, 85, 57	Fatty acid	^52^
13	13.51	Nonatriacontanoic acid	C_39_H_78_O_2_	577.5	–	95, 81, 71	Fatty acid	^53^
14	13.69	Tri-caffeoyl-anhydro-octulopyranosonic acid	C_35_H_30_O_17_	721	–	397, 277, 118	Phenolic acid	^44^
15	14.05	Hydroxy octadecatrienoic acid	C_18_H_30_O_3_	293	317 [M + Na]	293, 275, 121, 97	Fatty acid	^54^
16	14.17	Hydroxy octadecatrienoic acid isomer	C_18_H_30_O_3_	293	317 [M + Na]	293, 275, 121, 97	Fatty acid	^54^
17	15.20	Hydroxyoleuropein	C_25_H_32_O_14_	555	579 [M + Na]	225, 207, 153, 135	Secoiridoid	^47^
18	17.94	Pheophorbide B	C_35_H_34_N_4_O_6_	–	607	547	Chlorophyll catabolite	^46^
19	18.17	Linoleamide	C_18_H_33_NO	–	280	263, 245, 95	Fatty amide	^56^
20	19.01	Hydroxypalmitic acid	C_16_H_32_O_3_	271	–	225	Fatty acid	^55^
21	19.38	Palmitamide	C_16_H_33_NO	–	256	102, 88, 43	Fatty amide	^57^
22	19.90	Oleamide	C_18_H_35_NO	–	282	265, 247, 240, 177, 149, 135	Fatty amide	^57^
23	20.34	Quercetin-hydroxy-methylglutaryl-hexoside	C_27_H_28_O_16_	–	609	464, 303	Flavonoid glycoside	^37^
24	20.91	Pheophorbide A	C_35_H_36_N_4_O_5_	–	593	533	Chlorophyll catabolite	^46^
25	22.07	Octadecanamide (Stearamide)	C_18_H_37_NO	–	284	130, 116, 102,71	Fatty amide	^58^

Compound (1) was identified as carbendazim and belongs to the benzimidazole class^33,34^. Moreover, the identified flavonoids were quercetin glucoside and quercetin–hydroxy-methyl glutaryl hexoside^35–37^. In addition, dimethoxy hydroxyacetophenone was among the acetophenones class^38^. Phenolic acids such as dicaffeoyl quinic acid, ethyl caffeate, ferulic acid pentosyl and tri-caffeoyl-anhydro-octulopyranosonic acid were identified^39–44^. Compound (10) belongs to the triterpenes class and was identified as zahnic acid^45^. Furthermore, chlorophyll catabolites such as pheophorbide B and pheophorbide A were identified^46^. Additionally, compound (17) was identified as hydroxyoleuropein and belongs to secoiridoids^47^. Fatty acids and fatty amides such as corchorifatty acid F, trihydroxyoctadecenoic acid, octadecadienoic acid ethyl ester, hydroxy eicosapentaenoic acid, nonatriacontanoic acid, hydroxy octadecatrienoic acid, hydroxypalmitic acid, linoleamide, palmitamide, oleamide and stearamide were identified^29,48–58^. The detailed fragmentation of the identified compounds belonging to the different classes is displayed in the Supplementary Materials.

### *In silico *pharmacokinetic study

The results revealed that the majority of the compounds exhibited high predicted gastrointestinal absorption due to their reasonable solubility except the compounds namely, quercetin glucoside 2 and nonatriacontanoic acid 13. Furthermore, the compounds namely, quercetin glucoside 2, corchorifatty acid F 6, trihydroxyoctadecenoic acid 7, octadecadienoic acid ethyl ester 9 and nonatriacontanoic acid 13 showed no predicted blood brain barrier permeability. In addition, all the tested compounds were devoid of inhibitory effect on CYP 3A4. The results are listed in the Supplementary Materials Table S5. Moreover, the BOILED-Egg chart was used to evaluate the predicted passive gastrointestinal absorption of the molecules and brain penetration (Fig. S2), where, the yellow area (yolk) shows the molecules dimethoxy hydroxyacetophenone 3, ethyl caffeate 5, hydroxy octadecatrienoic acid 15, linoleamide 19, palmitamide 21, oleamide 22 and stearamide 25 possessing high probability to penetrate the brain (BBB). Furthermore, the white region shows the molecules corchorifatty acid F 6, trihydroxyoctadecenoic acid 7 and octadecadienoic acid ethyl ester 9 possessing high probability of passive gastrointestinal absorption (HIA). In the present study, it was observed that 10 compounds out of 13 were present in the prediction area. The compounds are illustrated in red color showing that they are non substrate of P-gp (PGP^-^) except compounds corchorifatty acid F 6 and trihydroxyoctadecenoic acid 7 expressed with the blue color as they are substrate for P-gp (PGP^+^).

### *In silico* molecular docking study

The major identified compounds by the UPLC-ESI–MS/MS were tested *in silico* against EGFR kinase (8A27), cyclin-dependent kinase 2 (1JSV) and VEGF-A (3QTK). All the tested compounds showed agreeable binding affinities to the tested targets with negative binding scores. It was observed that quercetin glucoside (2) exhibited the highest activity compared to the other compounds against EGFR with binding score = -10.1 kcal/mol comparable to the standard drug erlotinib eliciting a ∆G = -9 kcal/mol. In addition, compound (2) exhibited a strong activity against CDK2 with ∆G = -8.1 kcal/mol which was higher than the standard drug roscovitine having ∆G = -7.6 kcal/mol. Moreover, the compound carbendazim (1) exhibited higher activity than the other tested compounds against VEGF-A with binding score equal to -6.7 kcal/mol which was comparable to the standard drug triamcinolone eliciting a ∆G = -8.5 kcal/mol. The binding scores and the interactions are listed in the Supplementary Materials Table S6 and illustrated in Figs. S3, S4, S5, S6, S7, S8, S9, S10 and S11.

## Discussion

By reviewing the literature, it was observed that *Corchorus* species were employed in several cultures as a medicine. *C. olitorius* was utilized in folk medicine in the treatment of chronic cystitis, dysuria, fever, cold, and tumors. Moreover, reports showed that the plant is rich with proteins, calories, fibers and anti-tumor agents^17^. Therefore, the current work was carried out to assess the potential antiproliferative activity of *C. olitorius* leaves growing in Egypt and to identify the phytochemicals responsible for the activity.

The results of the in *vitro* assay revealed that the ethyl acetate fraction prepared from the 70% ethanol extract of *C. olitorius* exhibited strong cytotoxic activity against A549 (adenocarcinoma human alveolar basal epithelial cells) with IC_50_ = 7.8 µg/mL. It is important to note that plant extracts with IC_50_ less than 20 µg/mL are considered to possess potent *in vitro* cytotoxic activity according to the criteria of the US National Cancer Institute (NCI)^59^. Moreover, the current study showed that the ethyl acetate fraction exerted potent anti-angiogenic activity at a concentration of 50 µg/mL. It is important to note that; angiogenesis is greatly involved in solid carcinogenesis due to the predominance of pro-angiogenic factors over anti-angiogenic factors leading to the formation of new and abnormal blood vessels which elicit the growth and metastasis of cancer cells. The vascular endothelial growth factor-A (VEGF-A) is considered to be an important angiogenic promoter and is believed to be a potential target for tumor chemoprevention and treatment^5,60^. In the light of these results, it was found reasonable to study the *in vivo* anti-neoplastic activity of the ethyl acetate fraction. Ehrlich Ascites Carcinoma (EAC) is a reliable model which is widely employed to evaluate tumor pathogenesis and to develop anticancer agents^61^. The findings of the *in vivo* study supported the potential anti-proliferative activity of the ethyl acetate fraction which significantly reduced the tumor volume in the treated mice with 180 mg/kg bw. Also, the combination treated group showed significant reduction in the tumor volume which was comparable to the doxorubicin treated group. Moreover, no significant change in the body weight and no mortality were observed in the ethyl acetate fraction treated group which prove the safety of the employed fraction. The histopathological study regarding the ehrlich tumor confirmed the improvement in animal groups treated with doxorubicin, ethyl acetate fraction and combination compared to the untreated control group. Furthermore, the ethyl acetate fraction showed protective effect on the heart as the untreated control and the doxorubicin treated groups showed mildly congested sub-pericardial blood vessels, while, the ethyl acetate fraction treated group exhibited average blood vessels. The findings validate the traditional use of *C. olitorius* in the relief of engorged blood vessels^18^.

The immunohistochemical study on caspase-3 and Ki-67 expression was carried out. Caspase-3 which belongs to the cysteine protease family, is believed to be an important mediator of apoptosis in cells exposed to radiotherapy, immunotherapy or cytotoxic drugs. It is also employed as a marker to evaluate the effectiveness of cancer treatment^62^. In addition, Ki-67 is a prognostic and predictive marker used for the diagnosis and treatment of cancer. Ki-67 is associated with the growth and proliferation of the tumor cells^63^. The findings revealed that the doxorubicin and the ethyl acetate fraction treated groups elicited moderate cytoplasmic reactivity regarding caspase-3, while the untreated control and the combination groups exhibited weak cytoplasmic reactivity. Regarding Ki-67 expression, the untreated control and the ethyl acetate fraction treated groups exhibited more than 75% expression in tumor cells. In addition, the doxorubicin treated group showed 25 to 50% expression of ki-67, while the combination treated group elicited 51 to 74% expression of Ki-67. Thus, the observed anti-neoplastic activity in the ethyl acetate fraction treated group may be attributed to apoptosis, while it may be attributed to decreased Ki-67 expression and reduced cell proliferation in the combination treated group.

Previous reports revealed that various bioactive metabolites are present in the plant such as cardiac glycosides, carotenoids, phenolics, fatty acids, polysaccharides, triterpenes, steroids, minerals, proteins and vitamins^13,19,20^. In the present study, Folin-Ciocalteu and aluminium chloride methods revealed the presence of phenolic and flavonoids compounds. Reports showed that the phenolic compounds possess potent anticancer activity through several mechanisms. They regulate the level of reactive oxygen species leading to decreased cell proliferation and apoptosis. Also, they increase tumor suppressor protein expression and decrease the level of oncogenic proteins^64^. Moreover, flavonoids possess potential anticancer activity by eliciting apoptosis and arresting the proliferation and metastasis of cancer cells^65^.

In the current study, the UPLC-ESI-MS/MS investigation of the ethyl acetate-soluble fraction revealed the presence of different bioactive compounds belonging to various chemical classes, such as benzimidazoles, flavonoids, acetophenones, phenolic acids, triterpenes, steroids, chlorophyll catabolites, secoiridoids, fatty acids, and fatty amides. The observed cytotoxic, anti-angiogenic, and antiproliferative activities of the fraction may be attributed to the synergistic action between the present compounds^66^. The *in silico* pharmacokinetic study on the major tentatively identified compounds showed safe pharmacokinetic profile. The predicted effect of the compounds on CYP3A4 was studied. CYP3A4 represents 30% of the total P450 quantity in the liver. A large number of anticancer medications are metabolized by CYP3A4. Patients suffering from cancer are administered combination remedy which may lead to possible drug-drug interactions, severe side effects, serious toxicities or could reduce drug’s effectiveness^67,68^. The tested compounds showed no effect on CYP3A4 eliminating any possible interactions or side effects. Moreover, the *in silico *pharmacodynamic study was carried out to evaluate the effects of the major identified compounds on possible molecular targets involved in cancer. The epidermal growth factor receptor (EGFR) belongs to tyrosine kinase family and is responsible for tumor growth and metastasis, therefore, it represents a potential therapeutic target^69^. Moreover, cyclin dependent kinase belongs to serine / threonine kinases and is related to cancer pathogenesis through increasing the proliferation of cancer cells and affecting cell cycle transition^70^. Furthermore, vascular endothelial growth factor (VEGF) inhibitors have been largely employed in the management of cancer^71^. VEGF-A is responsible for inducing angiogenesis and promoting the proliferation and metastasis of endothelial cells^72^. Results of the *in silico* study revealed that all the tested compounds possessed promising binding affinities toward the tested receptors displaying negative binding scores comparable to the tested standard drugs.

Reports revealed that the tentatively identified compound carbendazim (1) which belongs to benzimidazole group possessed potent cytotoxic activity against different human cell lines due to its ability to inhibit tumor cell proliferation and was subjected phase 1 clinical trials^73^. Moreover, the compound quercetin glucoside (2) was previously identified in *C. olitorius* leaves growing in Tunisia^28^. By reviewing the literature, it was observed that the compound exhibited strong antiproliferative activity against HepG-2 cell lines by several mechanisms such as activation of caspase-3 leading to induction of apoptosis. Moreover, it induced cell cycle arrest at S phase. In addition, it inhibited DNA topoisomerase II^74^.

Previous reports showed that the phenolic compound ethyl caffeate exerted potent cytotoxic activity against ovarian cancer cell lines (SKOV-3) by decreasing cell proliferation. Also, it inhibited cyclin dependant kinase expression leading to cell cycle arrest at G1 phase^75^. Moreover, polyunsaturated fatty acids fall under the group of safe nutraceutical agents that could be used in cancer treatment. Reports showed that the conjugated eicosapentaenoic acid, gamma-linolenic acid (6,9,12, octadecatrienoic acid) and its derivatives possessed strong antineoplastic activity through several mechanisms. It was observed that the conjugated eicosapentaenoic acid inhibited the proliferation of cancer cells and reduced DNA polymerase and topoisomerase activities. In addition, gamma linolenic acid and its derivatives exhibited anti-angiogenic and antiproliferative activities^76^. Furthermore, fatty acid amides play a role as anticancer agents by affecting the proliferation of cancer cells^77^. It was reported that oleamide (22) exhibited potent antineoplastic activity by decreasing the expression of Bcl-2 and increasing caspase-3 activation leading to apoptosis. Also, it causes cell cycle arrest^78^. Moreover, reports revealed that pheophorbide A possessed potent *in vivo* antitumor activity against lung and liver cancers^79^.

The *in vitro* and *in vivo* findings support the potent anticancer and anti-angiogenic activities of *C. olitorius* leaves growing in Egypt which could be attributed to its chemical composition. Therefore, further in depth studies should be carried out on this plant to isolate the bioactive compounds which could represent promising candidates for developing effective chemopreventive and anticancer agents.

## Materials and methods

### Plant material

The fresh leaves of *C. olitorius* L. growing in Egypt were collected in March 2021 from the Zoo, Giza, Egypt (approximate coordinate: 30°1′28.32’’N, 31°12′50.03’’E). The plant collection adhered to the national and international legislation concerning the use of plant material for scientific research. As *C. olitorius* is a commonly cultivated species and not listed under any national or international conservation regulations, no special permits were required for its collection or use. We confirm that the current study did not involve any endangered or protected plant species. The collection site features loamy, well-drained soil with regular irrigation. The collection area is exposed to partial to full sunlight. The environmental conditions at the time of harvest were in accordance to the region’s semi arid climate, with mild temperature and low level of humidity. No pesticide or herbicide treatments were observed at the collection site during the collection process. The plant was kindly authenticated by Mrs. Therease Labib, consultant of plant taxonomy at the Ministry of Agriculture, Egypt and the former director of El-Orman Botanical Garden (Taxonomist). A voucher specimen (PHG-P-CO-338) was deposited at the botanical herbarium of the Pharmacognosy Department, Faculty of Pharmacy, Ain Shams University, Cairo, Egypt. The specimen is available for reference upon request.

### Preparation of the plant extract

The air-dried leaves (3 kg) were milled and extracted three times by maceration with 70% distilled ethanol with occasional shaking. Each maceration cycle was performed over a period of seven days. A total of 20 L of 70% distilled ethanol were used during the 3 extraction cycles. After each extraction cycle, 70% distilled ethanol was filtered, and fresh 70% ethanol was added to the next cycle. The ethanol extract was evaporated under reduced pressure at 45 °C using a rotary vacuum evaporator. The obtained residue (190 g) was dissolved in 70% ethanol and then the extract was fractionated using solvents with different polarities. The extract was shaken with distilled *n*-hexane (14 L) to obtain a *n*-hexane fraction of 55 g. Immediately after obtaining the *n*-hexane fraction, the remaining 70% ethanol was concentrated to dryness under reduced pressure at 45 °C without any intermediate processing steps. The obtained residue was redissolved in 100% distilled water and then shaken with ethyl acetate (19 L) to yield the ethyl acetate fraction (20 g). The remaining 100% water fraction was lyophilized to yield 70 g. A detailed flow diagram of the fractionation process is provided in the Supplementary Materials (Fig. S12).

The primary solvent used for extraction was 70% ethanol owing to its ability to efficiently dissolve a broad range of high- and moderate polar metabolites. Moreover, the selection of solvents for subsequent fractionation was based on their varying polarities, allowing for the targeted separation of different classes of secondary metabolites. A non-polar solvent such as *n*-hexane was used to obtain lipophilic compounds, while ethyl acetate, with intermediate polarity, was suitable for moderately polar compounds. In addition, water was used to extract highly polar metabolites^80,81^.

The yield percentage of each fraction was determined as the weight of the respective fractions divided by the weight of the crude ethanol extract and multiplied by 100. The *n*-hexane fraction yielded 28.95%, the ethyl acetate-soluble fraction yielded 10.53%, and the water fraction yielded 36.84%.

The obtained plant extract and fractions were subjected to *in vitro* biological screening to detect the most bioactive extract and fractions. The ethyl acetate-soluble fraction was the most bioactive. Therefore, it was subsequently used for metabolite profiling using UPLC-ESI-MS/MS analysis, as detailed in the section material and methods (phytochemical analysis, UPLC-ESI-MS/MS analysis).

### *In vitro determination of the cytotoxic activity*

#### Cell culture

Determination of the cytotoxic activity of 70% ethanol extract of *C. olitorius* as well as the prepared fractions was carried out on A549 (adenocarcinoma human alveolar basal epithelial cells), HepG2 (human liver cancer cell line) and MDA-MB-231 (invasive ductal carcinoma). The cell lines were purchased from American Type Culture Collection (ATCC; Manassas, VA, USA). Dulbecco’s modified Eagle’s medium–high glucose powder having 10% heat-inactivated fetal bovine serum, 1 mM sodium pyruvate, 100 µg/mL streptomycin, 100 U/mL penicillin and 2 mM L-glutamine was employed to preserve the cells. Culture dishes (Cellstar) containing the cells were stored in a humidified chamber at 35 °C. The chamber was provided with 5% (v/v) CO_2_. Finally, the cells were kept as a monolayer culture with serial subculturing. Logarithmic phase growing cells were utilized in all experiments^82^.

Assessment of the cytotoxic activity.

The cytotoxic activity of the total extract and fractions was tested against the mentioned cell lines using MTT (3-[4,5-dimethylthiazole-2-yl]-2,5-diphenyltetrazolium bromide) assay^83,84^. Cell suspensions were trypsinized, counted and seeded into 96-well microliter plates. Overnight incubation of the cells was carried out. The tested extract and fractions were dissolved in dimethyl sulfoxide to prepare stock solutions (1 mg/mL). Following this, cells were exposed to the extract and fractions at a concentration of 20 µg/mL for 72 h. Then, the growth media was removed; cells were incubated with 100 μL of MTT solution/well and were allowed to metabolize the dye into colored-insoluble formazan crystals for 1 h. The remaining MTT solution were discarded from the wells and the formazan crystals were dissolved in DMSO. Absorbance was measured at 550 nm. Doxorubicin was the standard control. Finally, the cell viability was calculated using the following equation:

$$\text{\% cell viability}=\frac{\text{O}.\text{D of treated cells}-\text{O}.\text{D of culture medium}}{\text{O}.\text{D of untreated cells}-\text{O}.\text{D of culture medium }}\text{ x }100$$

O.D = optical density.

### Assessment of the anti-angiogenic activity

The endothelial progenitor cells (EPCs) were cultured with a density of 5 × 10^3^ cells/ well in 96-well plates. After one day incubation, the culture medium was removed and replaced with fresh MV2 complete medium. DMSO was used as a control. The extract and fractions were tested at a concentration of 50 µg/ml. The cells were treated for 48 h. The treatment ended by the addition of 50% TCA. Incubation of the plate with 0.4% SRB in 1% acetic acid was carried out for 15 min. Finally, removal of excess solution was carried out and the dye was solubilised with 10 mM Tris buffer. Absorbance was measured using an ELISA reader at 515 nm^85^.

### *In vivo study*

#### Experimental animals

Thirty-four BALB/ c female mice (22–30 g, 6–7 weeks old) were used. Mice were supplied from the animal facility of the British University in Egypt. All animals were kept under standard hygienic conditions. Balanced diet was supplied to mice.

All animal procedures were approved by the ethics committee for Experimental, Clinical and Chemical Studies at Faculty of Pharmacy-The British University in Egypt under protocol number [EX-2410], and we confirm that all methods were carried out in strict accordance with the international ethical guidelines for the use of animals in research. Animals enrolled in the study were treated in compliance with the standards set forth by the National Institutes of Health on the care and use of laboratory animals (8^th^ edition), and the European Union Directive 2010/63/EU on the protection of animals used for scientific purposes. The study was carried out as per the ARRIVE guidelines^86^.

#### Ehrlich ascites carcinoma cells model

Ehrlich Ascites Carcinoma cells (EAC) were introduced intraperitoneally (I.P) in two mice in order to elicit ascites. Aspiration of the ascetic fluid was carried out after 7 days. The fluid was diluted 1:10 in saline and introduced intramuscularly (I.M) into the right flank of 32 mice to produce a solid tumor mass. On the 10^th^ day post tumor’s implantation, mice were divided into 4 groups, when the tumors were detectable in mice thighs.

Group I (control): untreated control group receiving saline, (*n* = 8).

Group II (Doxorubicin): Received 2 mg/kg bw doxorubicin I.P three times a week (*n* = 8).

Group III (EtOAC fraction): Received 180 mg/kg bw of *C. olitorius* ethyl acetate fraction I.P three times a week (*n* = 8).

Group IV (Combination): Received 2 mg/kg bw doxorubicin and 180 mg/kg bw *C. olitorius* ethyl acetate fraction I.P three times a week (*n* = 8).

The weight of the mice was recorded three times a week using a digital balance, and any mortality incidence was reported^87^. Moreover, measurement of the tumor dimensions (length and width) was carried out two times a week by employing a digital caliper to calculate the tumor volumes using the following equation:

Tumor volume (mm^3^) = 0.52 x (minor axis)^2^ x (major axis).

At the 26^th^ day post tumor implantation, mice were anaesthetized using sodium pentobarbital (50 mg/kg I.P) and sacrificed by cervical dislocation and tumors were collected and divided into 2 parts. One part was kept at − 20 °C until further processing. Moreover, the other part was kept in tubes containing 10% formalin-saline solution for histological study.

#### Histopathological examination

The excised tumor tissues and hearts were collected from the mice of the four groups and fixed in 10% neutral buffered formalin solution. Dehydration of the fixed tumors and hearts was carried out using ethanol, cleared in xylene, then placed in paraffin wax for investigation. Finally, the tumors and hearts were cut into thin 4 µm sections, de-waxed, rehydrated, then, stained with hematoxylin and eosin and examined with light microscopy in order to investigate their structures^88^.

#### Immunohistochemical study on the tumor tissues

The tumor sections were deparaffinised and rehydrated. Incubation of the tissues with citrate buffer for antigen retrieval was carried out. Then, the tumor sections were blocked using 3% hydrogen peroxide solution. Afterwards, incubation of the slides was carried out overnight in a humidified chamber by using primary antibodies against the active caspase-3 in a dilution of 1: 100 (ABclonal, USA, A2156, IgG) or Ki-67 in a dilution of 1:100 (QR015, IgG). Following this, horseradish peroxidase–conjugated sterptavidin was employed to incubate the slides. Diaminobenzidine reagent was employed followed by counter-staining with haematoxylin in order to visualize the immune reactions using light microscopy^89^.

#### Statistical analysis

Values were expressed as means ± standard deviation (SD). Results were analyzed using Two-way analysis of variance (ANOVA) followed by Tukey–Kramer test for *post hoc* analysis. Statistical results were determined to be significant at a *p*-value of less than 0.05. All statistical analyses were performed using GraphPad prism software. Graphs were plotted using GraphPad Prism software, version 10.1.2 for Windows (GraphPad Software, Inc. La Jolla, CA, USA).

### Phytochemical analysis

#### Determination of the total phenolic contents

Stock solutions of the standard gallic acid (1 mg/mL) and the ethyl acetate fraction (7.5 mg/mL) were prepared in methanol. Different dilutions (200, 400, 600, 800 and 1000 µg/mL) were prepared from the gallic acid stock solution. Folin-Ciocalteu method was used to assess the total phenolic content^90^. The prepared gallic acid and ethyl acetate fraction solutions were transferred to a 96-well microplate and each solution (10 µL) was mixed with 100 µL of Folin-Ciocalteu reagent. Following this, 80 µL of 1 M Na_2_CO_3_ was added and incubation in the dark was carried out at 25 °C for 20 min. Absorbance was measured at the end of the incubation time at 630 nm using a FluoStar Omega microplate reader. The results are displayed as means ± SD.

#### Determination of the total flavonoid contents

Stock solutions of the standard rutin (200 µg/mL) and the ethyl acetate fraction (7.5 mg/mL) were prepared in methanol. Different dilutions (7.8, 15.6, 31.2, 62.5, 125, 250, 500 and 1000 µg/mL) were prepared from rutin stock solution. Aluminum chloride method with minor modifications was employed to determine the total flavonoid contents^91^. The prepared rutin and ethyl acetate fraction solutions were added in a 96-well microplate and each solution (15 µL) was mixed with 175 μL of methanol. Then, 30 μL of 1.25% AlCl_3_ was added. At the end, 30 μL of 0.125 M C_2_H_3_NaO_2_ was added. Incubation was carried out for 5 min. Absorbance was measured at the end of the incubation time at 420 nm using a FluoStar Omega microplate reader. The results are presented as means ± SD.

#### UPLC-ESI-MS/MS analysis

The bioactive ethyl acetate-soluble fraction of *C. olitorius* leaves was used as the target for metabolic profiling. The sample was diluted to a final concentration of 100 μg/mL using HPLC-grade methanol. The sample was filtered using (0.2 μm) membrane disc filter and degassed via sonication prior to injection into UPLC-ESI–MS/MS. The sample was analyzed using UPLC-ESI–MS/MS at the Center for Drug Discovery, Research and Development, Faculty of Pharmacy, Ain Shams University. Ten microliters (10 μL) of the sample was injected into a Waters Xevo TQD mass spectrometer (Milford, MA 01757, USA) equipped with a reverse-phase C_18_ column (ACQUITY UPLC BEH C_18_, 1.7 μm, 2.1 × 50 mm). Gradient elution was employed. The mobile phase consisted of two solvents. Eluent A was H_2_O acidified with 0.1% formic acid and eluent B was acetonitrile acidified with 0.1% formic acid. The flow rate was 0.2 mL/min. The elution was as follows: (10% B) from 0 to 5 min.; (30% B) from 5 to 15 min.; (70% B) from 15 to 22 min.; (90% B) from 22 to 25 min. and (100% B) from 25–29 min. Both positive and negative ion modes were used in the analysis to achieve comprehensive metabolite profiling as follows: source temperature, 150 °C; cone voltage, 30 eV; capillary voltage, 3 kV; cone gas flow, 50 L/h; desolvation gas flow, 900 L/h; and desolvation temperature, 440 °C. Mass spectra were detected in the ESI-positive and -negative ion modes between *m/z* 50–2000. Plant extracts contain a wide variety of metabolites possessing different chemical properties. The employment of the dual-mode approach accounts for the diverse chemical nature of plant-derived compounds, as certain metabolites ionize preferentially in either the positive or negative mode depending on their functional groups and polarity. MassLynx 4.1 software was used to process the peaks and spectra. The compounds were tentatively identified through the comparison of their exact mass and MS/MS spectra with previous reported literature on *Corchorus* species and online databases (MassBank)^92^.

#### *In silico* pharmacokinetic study

The pharmacokinetic profiles of the major compounds identified by UPLC-ESI–MS/MS analysis were predicted using the SWISS ADME tool (www.swissadme.ch), a free web resource for evaluating ADME properties. Absorption, distribution, and metabolism were predicted. Moreover, the BOILED-Egg chart revealed important pharmacokinetic parameters, such as blood brain barrier permeability (BBB), passive gastrointestinal absorption (HIA), and P-glycoprotein substrate^93,94^.

#### *In silico* molecular docking study

Molecular docking of the major compounds identified by UPLC-ESI-MS/MS analysis was performed using the AutoDock Vina (1.2.0) platform^95^. The crystal structures of the targets EGFR kinase having the PDB ID (8A27), cyclin-dependent kinase 2 (CDK2) (1JSV), and VEGF-A (3QTK) were obtained from the Protein Data Bank (www.rcsb.org)^96^. The selection of the three target proteins was based on their pivotal roles in cell cycle regulation, tumorigenesis, and angiogenesis, which are central to the objectives of this work. Investigating these proteins provides valuable insights into the potential therapeutic effects of natural compounds in the management of cancer^69,71,72^. Furthermore, FDA-approved drugs targeting the EGFR kinase receptor are available; however, first-generation drugs like erlotinib showed limited effectiveness because of the development of resistance from mutations in the EGFR kinase receptor. In addition, second- and third-generation drugs are associated with significant side effects. As a result, it exists a need to discover safer and more effective alternatives^26,97^.

While performing docking, the exhaustiveness parameter was set to 8, the number of binding modes to 9, and the energy range to 3 kcal/mol. Three independent docking runs were conducted to ensure reproducibility. The grid box was centered on the EGFR kinase receptor at coordinates x = 38.72, y = 4.27, z = 57.97, with dimensions of 36.24°A × 28.90°A × 21.09°A, while in the case of CDK2, The grid box was centered at coordinates x = 17.74, y = 4.51, z = 24.68, with dimensions of 31.41°A × 27.03°A × 18.36°A. In addition, The grid box was centered at coordinates x = 47.76, y = 8.89, z = −18.43, with dimensions of 20.77°A × 23.98°A × 25.12°A in the VEGF-A receptor. Erlotinib, roscovitine, and triamcinolone were used as reference drugs against the tested targets, respectively. Erlotinib functions as an ATP analog, competing with ATP for binding to tyrosine kinase receptors, thereby inhibiting cell proliferation, inducing cell cycle arrest, and promoting apoptosis. Additionally, roscovitine inhibits cyclin-dependent kinases (CDKs) by directly competing with ATP at the binding site. Furthermore, studies have demonstrated that triamcinolone exhibits potential anti-angiogenic activity^98–100^. The chemical structures were drawn using ChemSketch 11.02. After structure drawing, the molecules were exported to Open Babel 3.1.1 to perform energy minimization, which was performed using the MMFF94 force field. The binding interactions were created using the Protein–Ligand Interaction Profiler web tool (http://plip-tool.biotec.tu-dresden.de)^101,102^. The 3D interactions were visualized using PyMOL 2.5.4. Hydrogen bonds and hydrophobic contacts were considered in particular. Hydrogen bonding significantly affects the solubility, distribution, and permeability of compounds, which are critical factors in drug effectiveness. Additionally, hydrogen bonds facilitate the binding between the drug and the receptor, improving the overall binding efficiency. Hydrophobic interactions, in turn, are vital for determining the binding affinity between the compound and the receptor. These interactions also contribute to the drug’s selectivity toward its target, which is essential for improving therapeutic efficacy. Understanding these interactions helps in interpreting the docking results and guides the optimization of drug candidates^103^. Validation of the docking was performed by docking the co-crystallized ligand in the receptor, then, calculation of RMSD by comparing the co-crystallized pose and the docked pose. A flow diagram of the overall docking workflow is provided in the Supplementary Materials (Fig. S13).

## Conclusions

Cancer represents a global health problem. Conventional chemotherapy has many drawbacks as they lack specificity, elicit many side effects, and is very expensive. Herbal nutraceuticals represent a pivotal source of bioactive compounds belonging to various phytochemical classes exhibiting potent pharmacological activities. *C. olitorius* is a herbaceous plant cultivated in many countries and was employed as a chief food in Egypt since the Pharaohs era as the leaves are rich in minerals and vitamins. Reports showed that the plant was widely used in folk medicine and exhibited a role in tumor management. The current study showed that the ethyl acetate-soluble fraction prepared from the 70% ethanol extract of *C. olitorius* leaves growing in Egypt possessed strong *in vitro* cytotoxic and anti-angiogenic activities. Moreover, the* in vivo *study proved the safety and the antiproliferative activity of the fraction against Ehrlich ascites carcinoma which consolidates its use in traditional medicine in tumor management. Furthermore, histopathological study showed that the fraction elicited protective effect on the heart. The observed activities were attributed to the bioactive metabolites present in the fraction belonging to different classes such as benzimidazoles, flavonoids, acetophenones, phenolic acids, triterpenes, steroids, chlorophyll catabolites, secoiridoids, fatty acids, and fatty amides. Therefore, *C. olitorius* leaves growing in Egypt represent a promising source of many leads from which effective chemopreventive and anticancer medications could be developed.

## Supplementary Information

Supplementary Information.

## Data Availability

Data presented in the study are available in this manuscript and Supplementary Materials. Further inquiries can be directed to the corresponding author.
