# Supplementary material for: Corchorus olitorius exhibits antiproliferative potential supported by metabolic profiling and integrative biological analyses
Source: Sci Rep. 2025 May 25;15:18166. doi: 10.1038/s41598-025-02717-1 (PMC12104385; doi:10.1038/s41598-025-02717-1)
Supplement: Supplementary file 1 — Supplementary Information. [file 41598_2025_2717_MOESM1_ESM.pdf]

Supplementary Materials

Corchorus olitorius exhibits antiproliferative potential supported by metabolic profiling and integrative biological analyses

Salma Sameh[1], Maha R. A. Abdollah[2], Ahmed M. Elissawy[1,3], Eman Al-Sayed[1], Rola M. Labib[1], Lan Ye[4], Fang-Rong Chang[5] , Abdel Nasser B. Singab[1,3]*

[1]Department of Pharmacognosy, Faculty of Pharmacy, Ain-Shams University, 11566, Cairo, Egypt

[2]Department of Pharmacology, Faculty of Pharmacy. The British University in Egypt, El Sherouk City, Egypt

[3]Center of Drug Discovery Research & Development, Faculty of Pharmacy, Ain-Shams University,11566, Cairo, Egypt

[4]Cancer Centre, the Second Hospital of Shandong University, Jinan, 250033, China. Address: 247 Beiyuan Street, Jinan, Shandong 250033, China

[5]School of Pharmacy and Graduate Institute of Natural Products, College of Pharmacy, Kaohsiung Medical University, Kaohsiung, 80708, Taiwan.

*Corresponding author

Professor Abdel Nasser B. Singab, Department of Pharmacognosy, Faculty of Pharmacy, Ain Shams University, 11566, Cairo, Egypt. Chairman of Center of Drug Discovery Research & Development, Faculty of Pharmacy, Ain-Shams University, 11566, Cairo, Egypt. Tel: +20224051120. Email: AbdelnasserSingab@pharma.asu.edu.eg, vpr.nassersingab@asu.edu.eg

List of Tables

Table S1. Cytotoxic activity of the 70 % ethanol extract of *C. olitorius* leaves and its subfractions.

Table S2. Anti-angiogenic activity of *C. olitorius* leaves.

Table S3. Effect of doxorubicin, EtOAC fraction and combination on tumor tissues in EAC bearing mice.

Table S4. Immunohitochemical study results on caspase-3 and Ki-67.

Table S5. Predicted pharmacokinetic profile of the major compounds identified in the ethyl acetate fraction of *C. olitorius* leaves.

Table S6. Binding interactions of the major compounds against EGFR kinase (8A27), (CDK2) (1JSV) and VEGF-A (3QTK)

List of Figures

Figure S1. a. ESI/MS positive ion mode chromatogram of the EtOAC fraction of *Corchorus olitorius*. b. ESI/MS negative ion mode chromatogram of the EtOAC fraction of *Corchorus olitorius*.

Figure S2. BOILED-Egg chart revealing the predicted absorption of the major compounds.

Figure S3. Three-dimensional interactions of compounds namely, carbendazim, quercetin hexoside, dimethoxy acetophenone, ethyl caffeate and corchorifatty acid F and the standard drug erlotinib with EGFR kinase (8A27) active sites.

Figure S4. Three-dimensional interactions of compounds namely, trihydroxyoctadecenoic acid, octadecadienoic acid ethyl ester, nonatriacontanoic acid, hydroxyl octadecatrienoic acid, linoleamide and palmitamide with EGFR kinase (8A27) active sites.

Figure S5. Three-dimensional interactions of compounds namely, oleamide and stearamide with EGFR kinase (8A27) active sites.

Figure S6. Three-dimensional interactions of compounds namely, carbendazim, quercetin hexoside, dimethoxy acetophenone, ethyl caffeate and corchorifatty acid F and the standard drug roscovitine with (CDK2) (1JSV) active sites.

Figure S7. Three-dimensional interactions of compounds namely, trihydroxyoctadecenoic acid, octadecadienoic acid ethyl ester, nonatriacontanoic acid, hydroxyl octadecatrienoic acid, linoleamide and palmitamide with (CDK2) (1JSV) active sites.

Figure S8. Three-dimensional interactions of compounds namely, oleamide and stearamide with (CDK2) (1JSV) active sites.

Figure S9. Three-dimensional interactions of compounds namely, carbendazim, quercetin hexoside, dimethoxy acetophenone, ethyl caffeate and corchorifatty acid F and the standard drug triamcinolone with VEGF-A (3QTK) active sites.

Figure S10. Three-dimensional interactions of compounds namely, trihydroxyoctadecenoic acid, octadecadienoic acid ethyl ester, nonatriacontanoic acid, hydroxyl octadecatrienoic acid, linoleamide and palmitamide with VEGF-A (3QTK) active sites.

Figure S11. Three-dimensional interactions of compounds namely, oleamide and stearamide with VEGF-A (3QTK) active sites.

Figure S 12. Flow diagram of the extraction and sequential solvent fractionation of *Corchorus olitorius* leaves.

Figure S 13. Overview of the molecular docking workflow used in this study.

Table S1. Cytotoxic activity of the 70 % ethanol extract of *C. olitorius* leaves and its subfractions

Cell line	Conc. µg/mL	% Cell inhibition			
		70% ethanol extract	*n*-hexane fraction	Ethyl acetate fraction	Aqueous residue
A549	20	22.1	41.4	81.2	3
HepG2	20	-20.6	8.9	12.3	-17.3
MDA-MB-231	20	17.6	29.7	44.9	16

Cytotoxic activity was tested at a concentration of 20 µg/ml and represented as percentage cell inhibition. Doxorubicin represented the positive control with IC_{50} = 0.86, 0.39, 0.94 µg/ml against A549, HepG2 and MDA-MB-231 cell lines, respectively

Table S2 . Anti-angiogenic activity of *C. olitorius* leaves

EPCs	70% ethanol extract	*n*-hexane fraction	Ethyl acetate fraction	Aqueous residue
% Cell survival	94	50	< 0	95

Table S3 . Effect of doxorubicin, EtOAC fraction and combination on tumor tissues in EAC bearing mice

	**Ehrlich tumor**						
	**Nodules**	**Tumor cells**	**Apoptosis**	**Mitosis**	**Karyorrhectic fragments**	**Necrosis**	**Others**
**Group I (Control)**	++	++	+	++	+	+	scattered giant cells
**Group II (Standard)**	+	+	0	0	0	0	-
**Group III (EtOAC fraction)**	++	+	+	0	+	0	-
**Group IV (Combination)**	+	+	0	0	+	0	-

Nodules: **0:** No nodules **+:** Small nodules **++:** Sheets/large nodules

Tumor cells: **0:** Non-viable **+:** Less-viable/mildly pleomorphic **++:** More-viable/markedly pleomorphic

Apoptosis: **0:** Marked **+:** Scattered **++:** No apoptosis

Mitosis: **0:** No mitosis **+:** Scattered **++:** Marked

Karyorrhectic fragments: **0:** Marked **+:** Few **++:** No

Necrosis: **0:** Large areas **+:** Small areas **++:** No necrosis

Table S4. Immunohitochemical study results on caspase-3 and Ki-67

	**Caspase-3**	**Ki-67**
**Group I (Control)**	+	+++
**Group II (Doxorubicin)**	++	+
**Group III (EtOAC fraction)**	++	+++
**Group IV (Combination)**	+	++

Caspase-3 reactivity was classified as: negative (0), weak (+), moderate (++), marked (+++).

Ki-67 positivity evaluated according to percentage of positive cells into four degree: (-) < 24%, (+) 25-50 % (Isolated), (++) 51-74% (Focal), (+++) > 75 % (Diffuse).

Table S5. Predicted pharmacokinetic profile of the major compounds identified in the ethyl acetate fraction of *C. olitorius* leaves

Compound	TPSA	Log P	Solubility	GI Absorption	BBB permeability	CYP 3A4 inhibition
Carbendazim (**1**)	67.01	1.09	Soluble	High	Yes	No
Quercetin glucoside (**2**)	210.51	0.94	Soluble	Low	No	No
Dimethoxy hydroxyacetophenone (**3**)	55.76	1.15	V. soluble	High	Yes	No
Ethyl caffeate (**5**)	66.76	1.82	Soluble	High	Yes	No
Corchorifatty acid F (trihydroxy octadecadienoic acid) (**6**)	97.99	2.78	Soluble	High	No	No
trihydroxyoctadecenoic acid (**7**)	97.99	2.83	Soluble	High	No	No
Octadecadienoic acid ethyl ester (**9**)	26.30	6.09	M. soluble	High	No	No
Nonatriacontanoic acid (**13**)	37.30	13.48	Insoluble	Low	No	No
Hydroxy octadecatrienoic acid (**15 – 16**)	57.53	4.31	Soluble	High	Yes	No
Linoleamide (**19**)	43.09	5.04	M. soluble	High	Yes	No
Palmitamide (**21**)	43.09	4.83	M. soluble	High	Yes	No
Oleamide (**22**)	43.09	5.32	M. soluble	High	Yes	No
Stearamide (**25**)	43.09	5.54	M. soluble	High	Yes	No

Table S6. Binding interactions of the major compounds against EGFR kinase (8A27), (CDK2) (1JSV) and VEGF-A (3QTK)

Compound Name	EGFR				CDK2				VEGF-A		
	Score	Hydrophobic	Hydrogen bond	Salt bridge	Score	Hydrophobic	Hydrogen bond	Salt bridge	Score	Hydrophobic	Hydrogen bond
Carbendazim (**1**)	-7.3	ALA 743	ASP 855	ASP 855	-6.6	ILE 10	LYS 33	-	-6.7	PHE 29	SER 43
		LYS 745	ASP 855			ILE 10	GLN 131			ILE 39	CYS 54
		THR 790	GLY 857			GLN 131	GLN 131			GLU 57	ASN 55
		LEU 844				ASN 132	ASP 145				ASP 56
											ASP 56
Quercetin glucoside (**2**)	-10.1	ALA 743	SER 720	-	-8.1	ILE 10	GLU 12	-	-6	-	LYS 41
		LEU 844	THR 854			VAL 18	ASP 86				ASN 55
		THR 854				ASP 86	GLN 131				ASN 55
							ASN 132				ASN 55
							ASP 145				HIS 79
											GLN 80
Dimethoxy hydroxyacetophenone (**3**)	-6.1	LEU 718	LYS 745	-	-5.9	ILE 10	LYS 33	-	-5.6	PHE 29	LYS 41
		VAL 726	THR 790			ILE 10				ILE 39	SER 43
		VAL 726	ASP 855			PHE 82				GLU 57	ASN 55
		ALA 743				LEU 134					ASP 56
		THR 790									
		LEU 792									
		LEU 844									
Ethyl caffeate (**5**)	-7.3	LEU 747	THR 854	-	-6.2	ILE 10	LYS 33	-	-5.9	ILE 22	LEU 25
		ILE 759	ASP 855			ASP 86	HIS 84			THR 24	LEU 25
		THR 790	PHE 856			LEU 134	ASP 86			THR 24	GLY 52
			GLY 857				ASP 86				
Corchorifatty acid F (trihydroxy octadecadienoic acid) (**6**)	-7.7	VAL 726	ALA 743	-	-5.5	ILE 10	ASP 86	-	-5.7	ILE 22	LEU 25
		ALA 743	LYS 745			VAL 18	GLN 131			ILE 22	LEU 25
		LYS 745	LYS 745			PHE 82				THR 24	LEU 25
		MET 766	THR 790			ASP 86				VAL 26	ASP 27
		LEU 777	THR 854			GLN 131				ARG 49	GLN 30
		LEU 777	ASP 855			LEU 134					
		LEU 788	ASP 855			LEU 134					
		LEU 788									
		LEU 788									
		THR 790									
		LEU 844									
		LEU 844									
Trihydroxyoctadecenoic acid (**7**)	-4.4	ALA 702	ARG 705	-	-5.6	VAL 18	GLU 12	-	-5.3	PHE 10	ILE 36
		ARG 705	ILE 706			ALA 31	GLU 12			PHE 10	GLU 37
						PHE 82	LEU 83			TYR 14	GLN 72
						GLN 131	GLN 131			ASP 34	LYS 100
											LYS 100
Octadecadienoic acid ethyl ester (**9**)	-3.9	TRP 731	-	ARG 776	-5.8	ILE 10	THR 14	LYS 129	-5.4	ILE 22	-
		ILE 740				ILE 10	THR 14			ILE 22	
		ILE 740				VAL 18				THR 24	
		LEU 778				ALA 31				ASP 27	
		GLN 791				PHE 82					
						ASP 86					
						LEU 134					
						LEU 134					
Nonatriacontanoic acid (**13**)	-6.4	PHE 723	TYR 891	-	-4.1	LEU 124	THR 182	-	-4.2	ILE 22	ASP 27
		PHE 723				LEU 124	THR 182			ILE 22	GLN 30
		VAL 726				LEU 124				THR 24	
		VAL 726				ARG 126				VAL 26	
		ALA 743				ARG 150				ASP 27	
		LYS 745				ARG 150					
		MET 766				LYS 178					
		LEU 777				TYR 179					
		LEU 788				TYR 179					
		THR 790				TYR 180					
		ARG 836									
		ASP 837									
		LEU 844									
		LEU 844									
		LEU 858									

Table S6. Binding interactions of the major compounds against EGFR kinase (8A27), (CDK2) (1JSV) and VEGF-A (3QTK) (Cont.)

Compound Name	EGFR				CDK2				VEGF-A		
	Score	Hydrophobic	Hydrogen bond	Salt bridge	Score	Hydrophobic	Hydrogen bond	Salt bridge	Score	Hydrophobic	Hydrogen bond
Hydroxy octadecatrienoic acid (**15-16**)	-7.3	VAL 726	ASP 855	-	-6.2	ILE 10	THR 14	-	-6	ILE 22	SER 43
		VAL 726				ILE 10	THR 14			ILE 22	CYS 54
		ALA 743				VAL 18	HIS 125			THR 24	
		LYS 745				VAL 18	ASP 127			LEU 25	
		LYS 745				ALA 31	LYS 129				
		LEU 777				LEU 134	ASN 132				
		LEU 788					ASP 145				
		THR 790									
		LEU 844									
		PHE 856									
Linoleamide (**19**)	-7.6	VAL 726	ASP 855	-	-5.1	ILE 10	GLN 131	-	-5.2	PHE 10	GLY 58
		ALA 743	ASP 855			ASP 86				TYR 14	
		LYS 745	ASP 855			LEU 134				GLU 37	
		LYS 745	PHE 856							LYS 100	
		MET 766	GLY 857								
		LEU 777									
		LEU 788									
		THR 790									
Palmitamide (**21**)	-6.9	LYS 745	MET 793	-	-5.1	ILE 10	HIS 84	-	-4.6	PHE 10	LYS 41
		LYS 745	MET 793			ILE 10				TYR 14	ASN 55
		LYS 745				VAL 18				ILE 36	
		CYS 775				VAL 18				ILE 36	
		LEU 777				ALA 31				LYS 100	
		LEU 777				PHE 82					
		LEU 788				LEU 134					
		THR 790									
		LEU 844									
Oleamide (**22**)	-7.2	LEU 718	THR 854	-	-5	ILE 10	LYS 89	-	-4.7	PHE 10	GLN 82
		VAL 726	ASP 855			ILE 10				TYR 14	
		VAL 726				ILE 10				ILE 36	
		LYS 745				VAL 18				GLU 37	
		LYS 745				PHE 82				LYS 100	
		LYS 745				LEU 134				LYS 100	
		LEU 777				LEU 134					
		LEU 788									
		THR 790									
		LEU 844									
		THR 854									
Stearamide (**25**)	-6.9	VAL 726	ARG 841	-	-5	ILE 10	GLU 8	-	-4.7	ILE 22	CYS 50
		VAL 726				ILE 10				ILE 22	
		ALA 743				ILE 10				THR 24	
		LYS 745				VAL 18				LEU 25	
		LYS 745				PHE 82					
		MET 766				GLN 131					
		LEU 777				LEU 134					
		LEU 788									
		LEU 844									
		PHE 856									
Erlotinib	-9	VAL 726	LYS 745	-	-	-	-	-	-	-	-
		ALA 743									
		LYS 745									
		LYS 745									
		LEU 788									
		THR 790									
		ARG 841									
		LEU 844									
Roscovitine	-	-	-	-	-7.6	ILE 10	GLU 12	-			
						ILE 10	LYS 33				
						VAL 18	GLN 131				
						ALA 31	GLN 131				
						ASP 86	GLN 131				
						ASN 132					
						LEU 134					
						LEU 134					

Table S6. Binding interactions of the major compounds against EGFR kinase (8A27), (CDK2) (1JSV) and VEGF-A (3QTK) (Cont.)

Compound Name	EGFR				CDK2				VEGF-A		
	Score	Hydrophobic	Hydrogen bond	Salt bridge	Score	Hydrophobic	Hydrogen bond	Salt bridge	Score	Hydrophobic	Hydrogen bond
Triamcinolone	-	-	-	-	-	-	-	-	-8.5	ILE 22	THR 24
										THR 24	LEU 25
											LEU 25
											GLY 52
											GLY 52

a

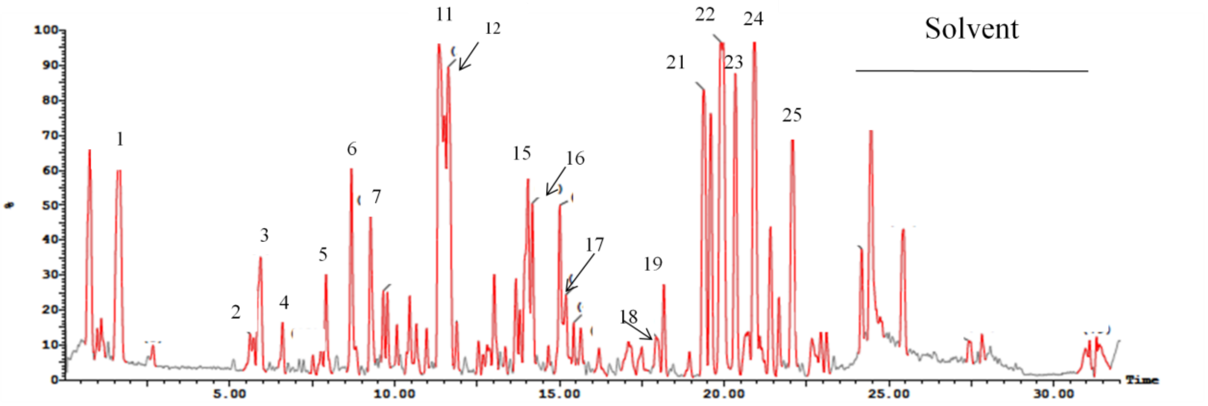

b

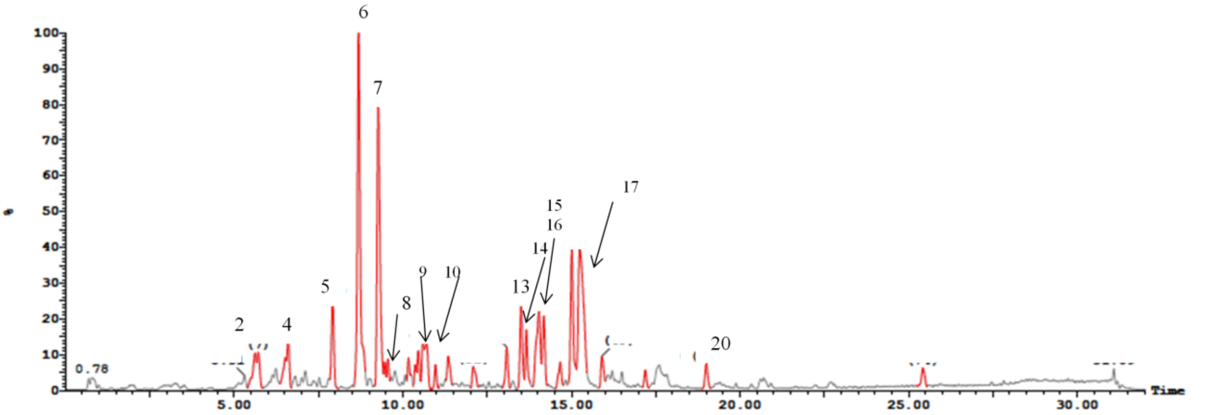

Figure S1. a. ESI/MS positive ion mode chromatogram of the EtOAC fraction of *Corchorus olitorius*. Major peaks are observed at retention times of 1.64 min $[M+H]^+$ 192, 8.69 min $[M+Na]^+$ 351, 9.28 min. $[M+Na]^+$ 353, 11.52 min. $[M+H]^+$ 319, 19.38 min. $[M+H]^+$ 256, 19.90 min. $[M+H]^+$ 282, 20.34 min. $[M+H]^+$ 609, 20.91 min. $[M+H]^+$ 593, 22.07 $[M+H]^+$ 284, corresponding to the compounds namely, carbendazim, corchorifatty acid F, trihydroxy octadecenoic acid, hydroxy eicosapentaenoic acid, palmitamide, oleamide, quercetin-hydroxy-methylglutaryl-hexoside, pheophorbide A and stearamide. b. ESI/MS negative ion mode chromatogram of the EtOAC fraction of *Corchorus olitorius*. Major peaks are observed at retention times of 8.69 min $[M-H]^-$ 327, 9.28 min. $[M-H]^-$329, corresponding to the compounds namely corchorifatty acid F, trihydroxy octadecenoic acid.

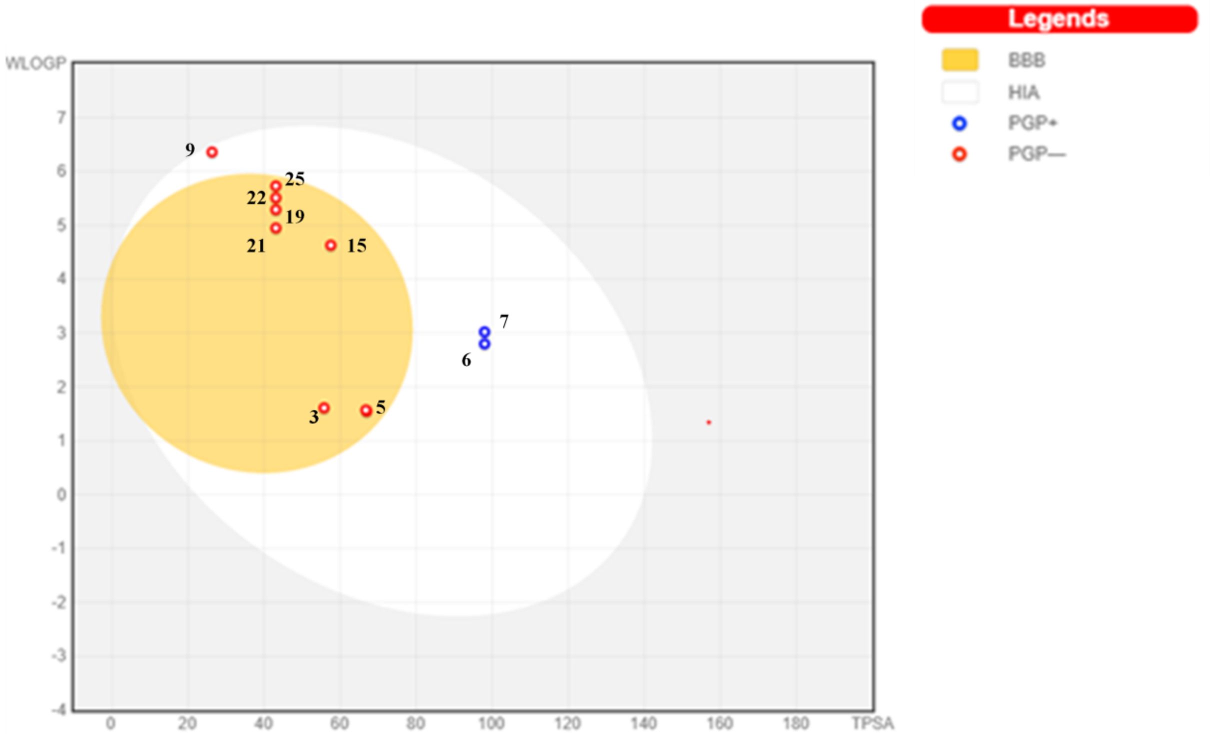

Figure S2. BOILED-Egg chart revealing the predicted absorption of the major compounds

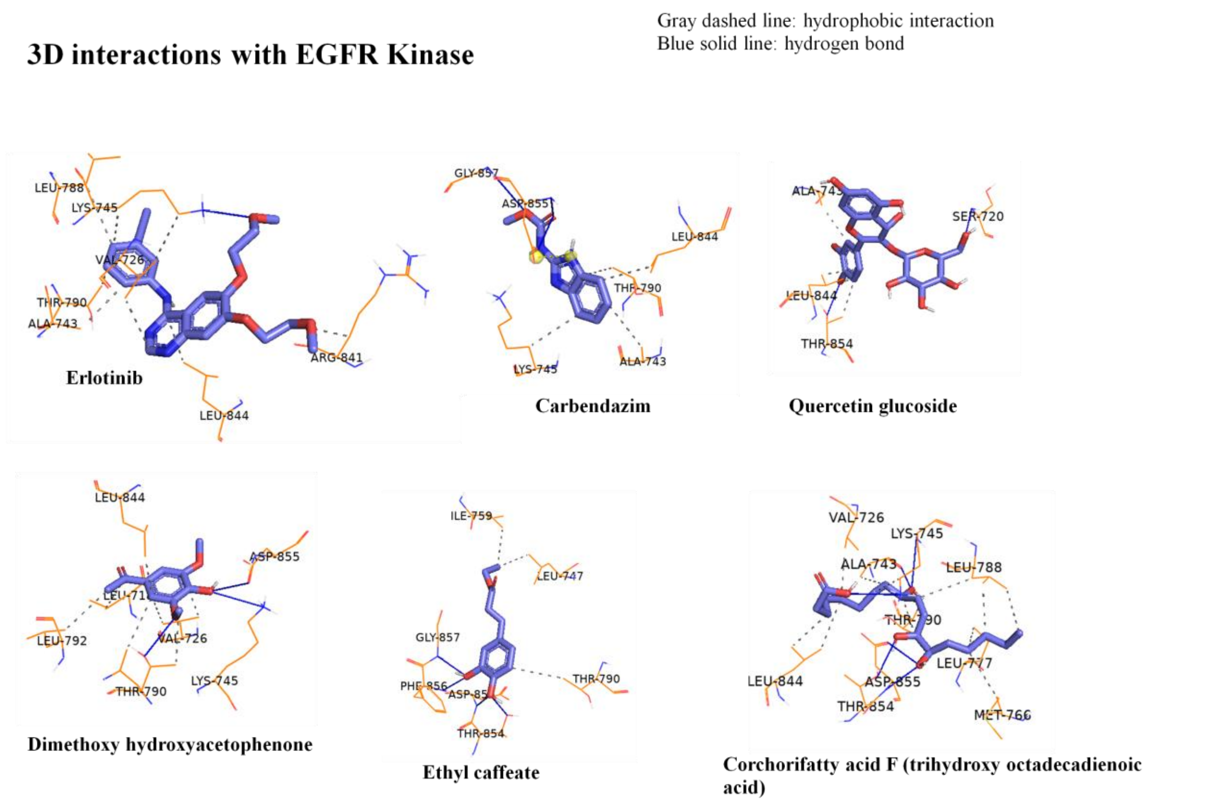

Figure S3. Three-dimensional interactions of compounds namely, carbendazim, quercetin glucoside, dimethoxy acetophenone, ethyl caffeate and corchorifatty acid F and the standard drug erlotinib with EGFR kinase (8A27) active sites

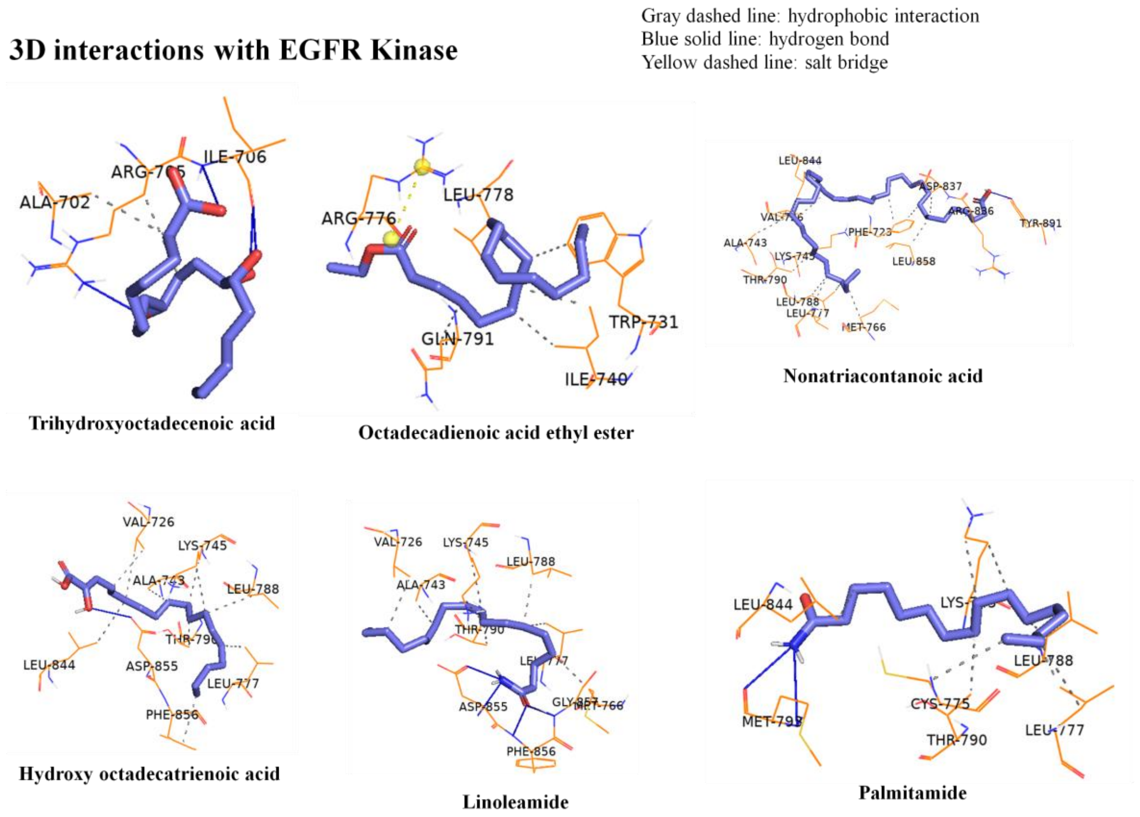

Figure S4. Three-dimensional interactions of compounds namely, trihydroxyoctadecenoic acid, octadecadienoic acid ethyl ester, nonatriacontanoic acid, hydroxy octadecatrienoic acid, linoleamide and palmitamide with EGFR kinase (8A27) active sites

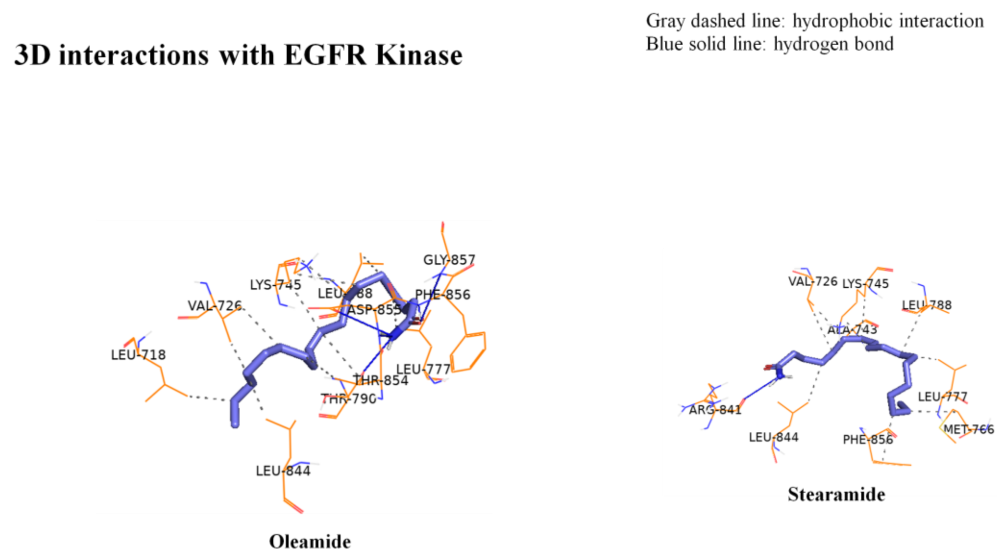

Figure S5. Three-dimensional interactions of compounds namely, oleamide and stearamide with EGFR kinase (8A27) active sites

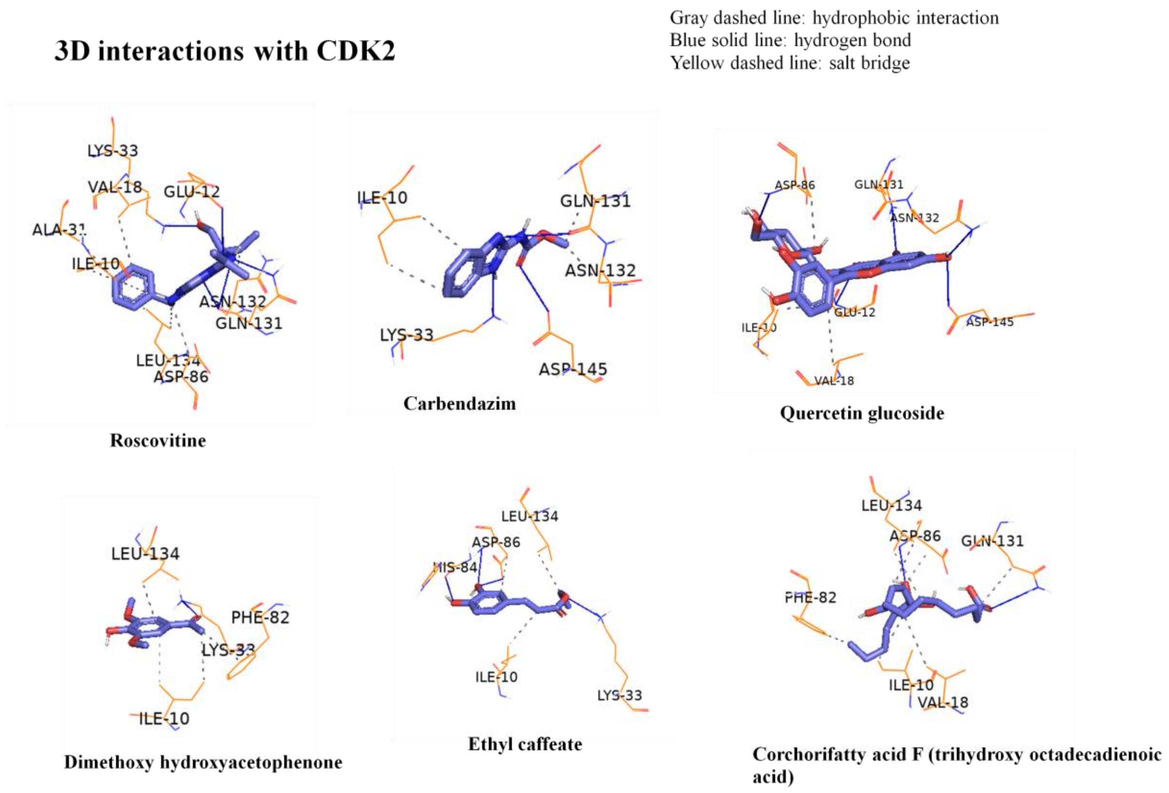

Figure S6. Three-dimensional interactions of compounds namely, carbendazim, quercetin glucoside, dimethoxy acetophenone, ethyl caffeate and corchorifatty acid F and the standard drug roscovitine with (CDK2) (1JSV) active sites

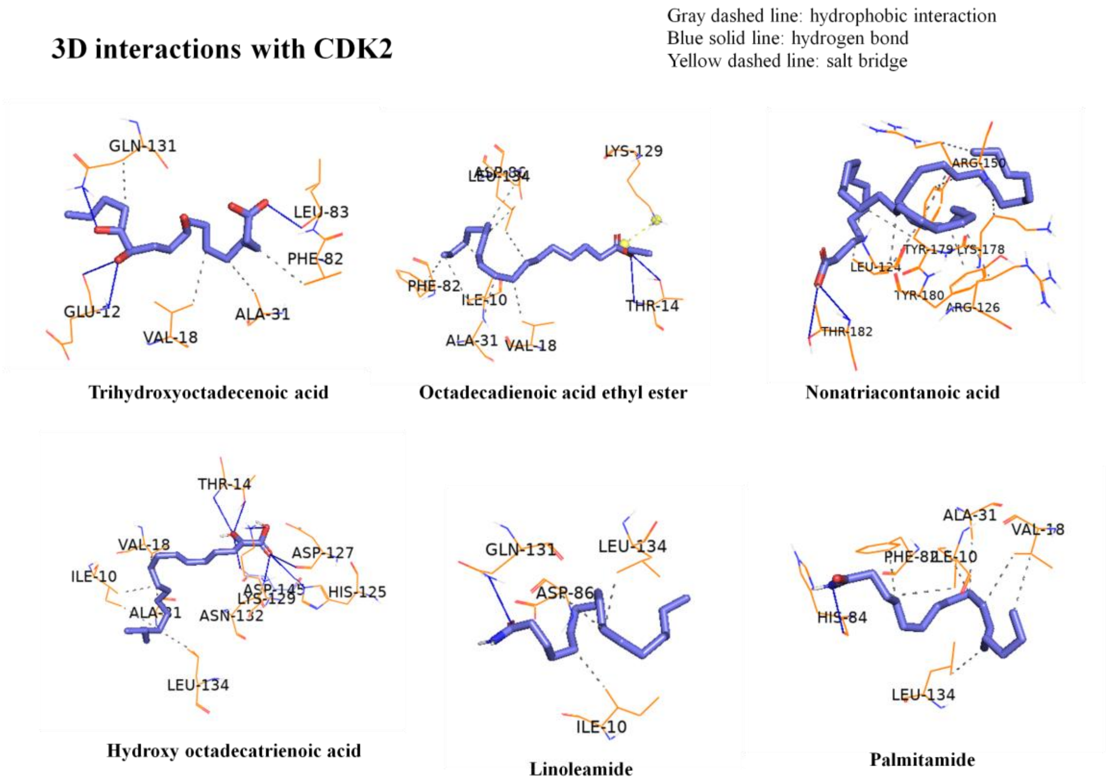

Figure S7. Three-dimensional interactions of compounds namely, trihydroxyoctadecenoic acid, octadecadienoic acid ethyl ester, nonatriacontanoic acid, hydroxy octadecatrienoic acid, linoleamide and palmitamide with (CDK2) (1JSV) active sites

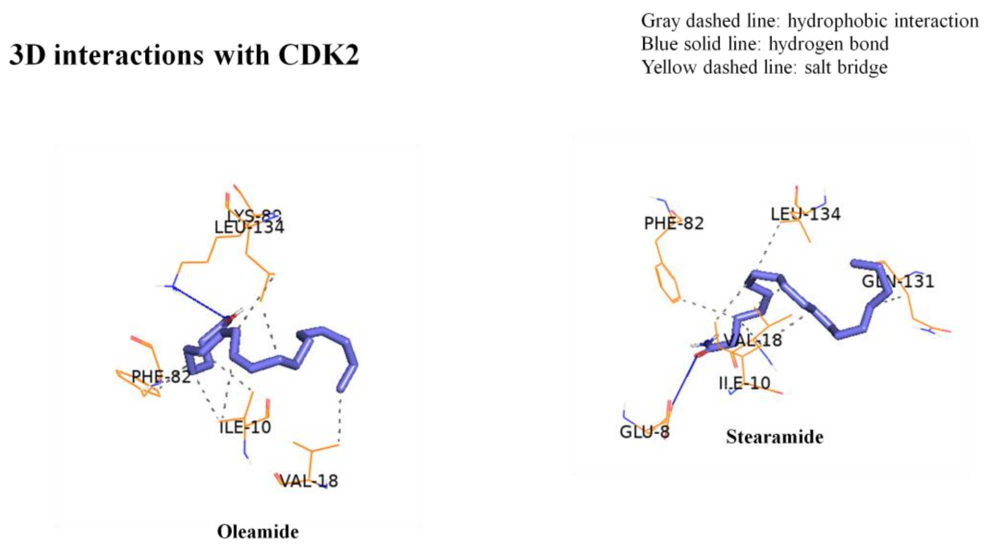

Figure S8. Three-dimensional interactions of compounds namely, oleamide and stearamide with (CDK2) (1JSV) active sites

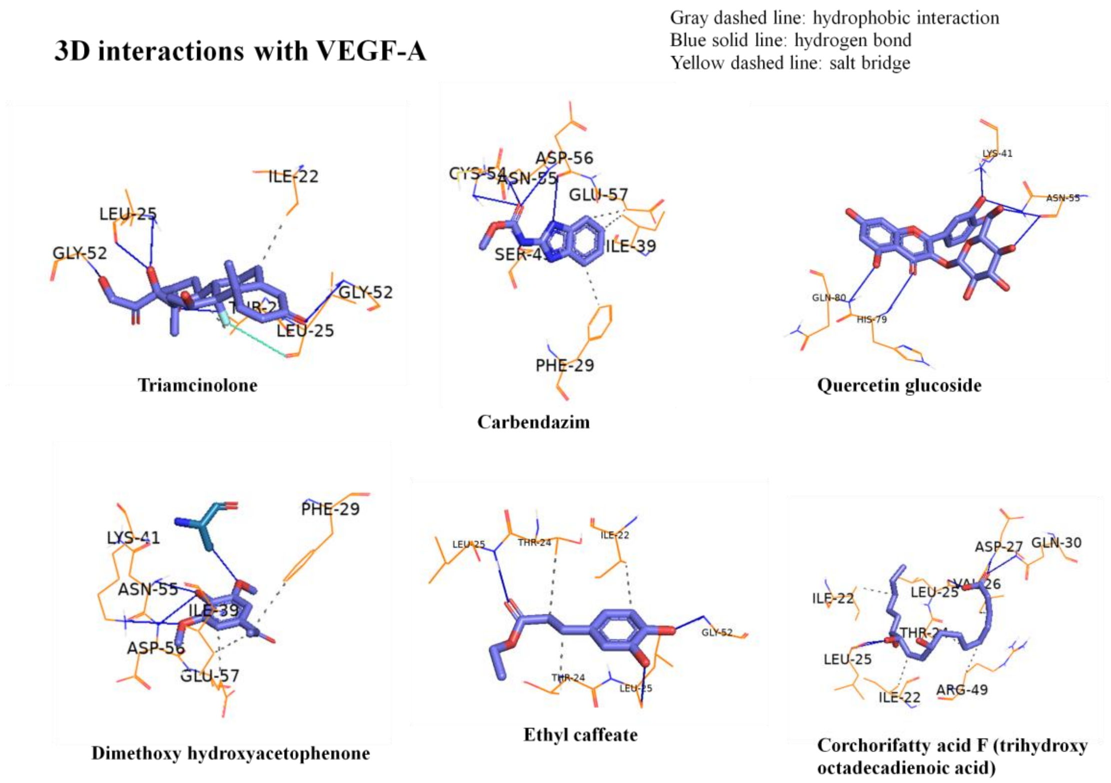

Figure S9. Three-dimensional interactions of compounds namely, carbendazim, quercetin glucoside, dimethoxy acetophenone, ethyl caffeate and corchorifatty acid F and the standard drug triamcinolone with VEGF-A (3QTK) active sites

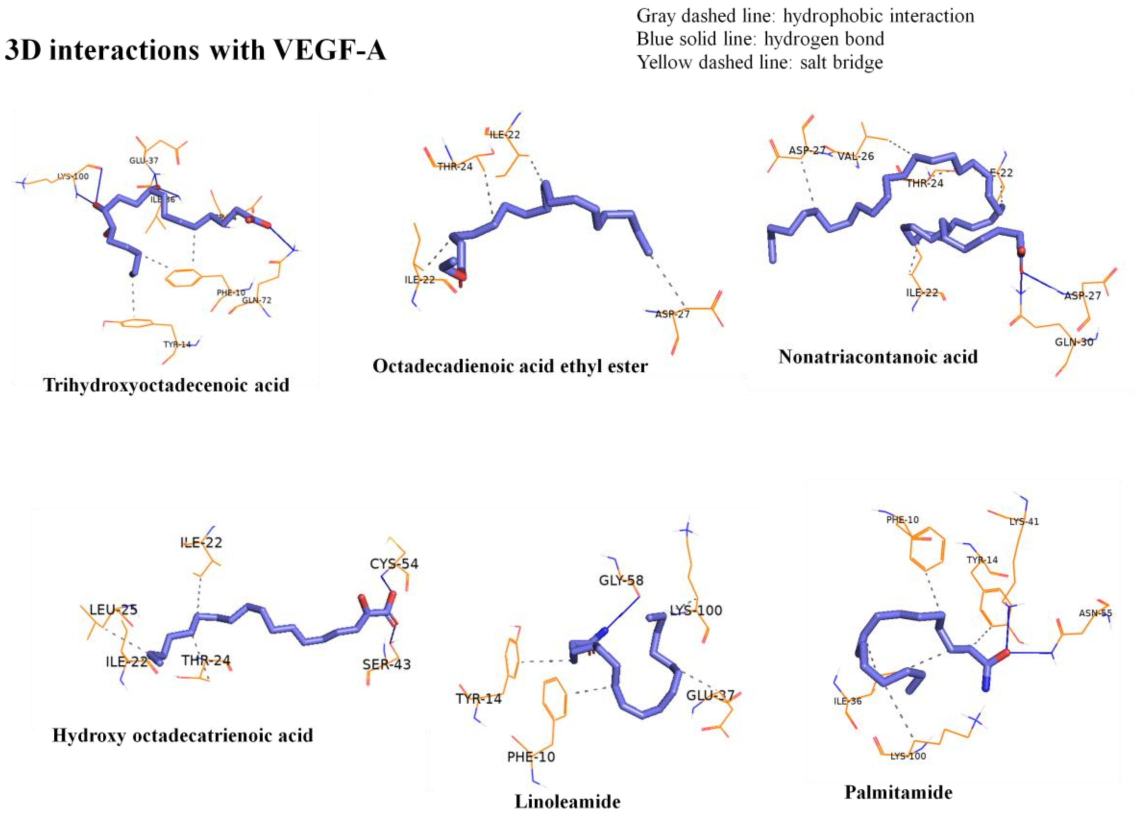

Figure S10. Three-dimensional interactions of compounds namely, trihydroxyoctadecenoic acid, octadecadienoic acid ethyl ester, nonatriacontanoic acid, hydroxy octadecatrienoic acid, linoleamide and palmitamide with VEGF-A (3QTK) active sites

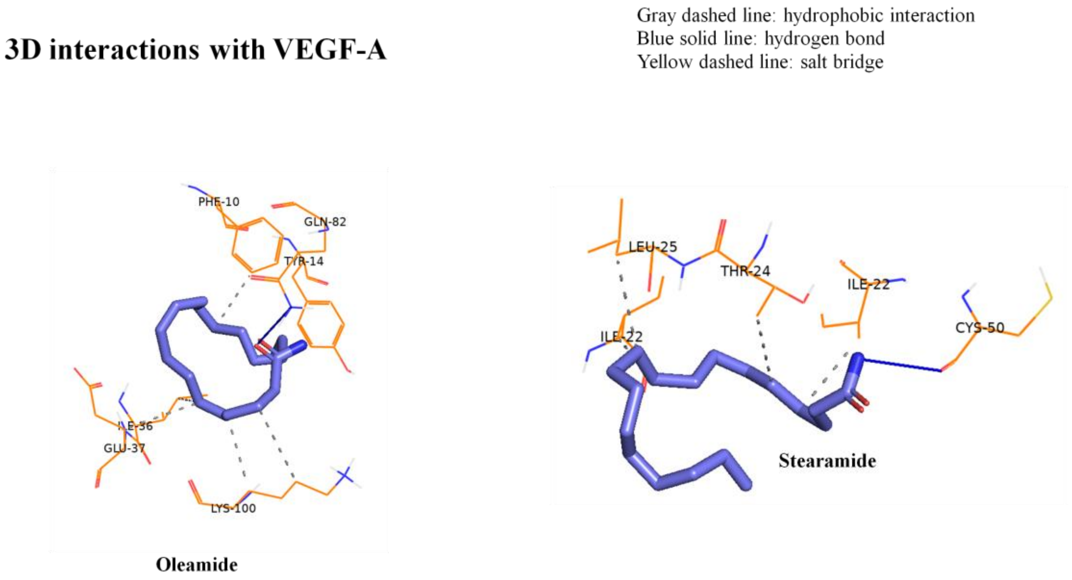

Figure S11. Three-dimensional interactions of compounds namely, oleamide, and stearamide with VEGF-A (3QTK) active sites

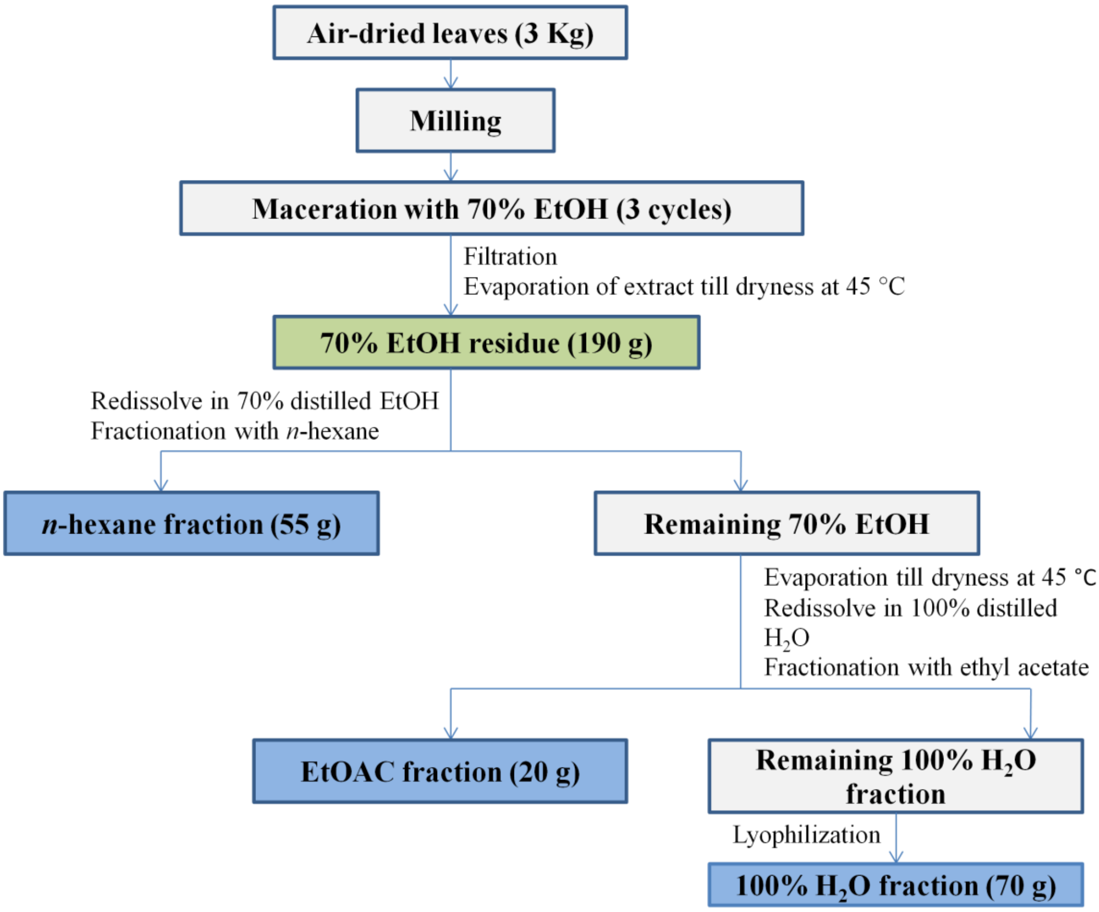

Figure S 12. Flow diagram of the extraction and sequential solvent fractionation of *Corchorus olitorius* leaves

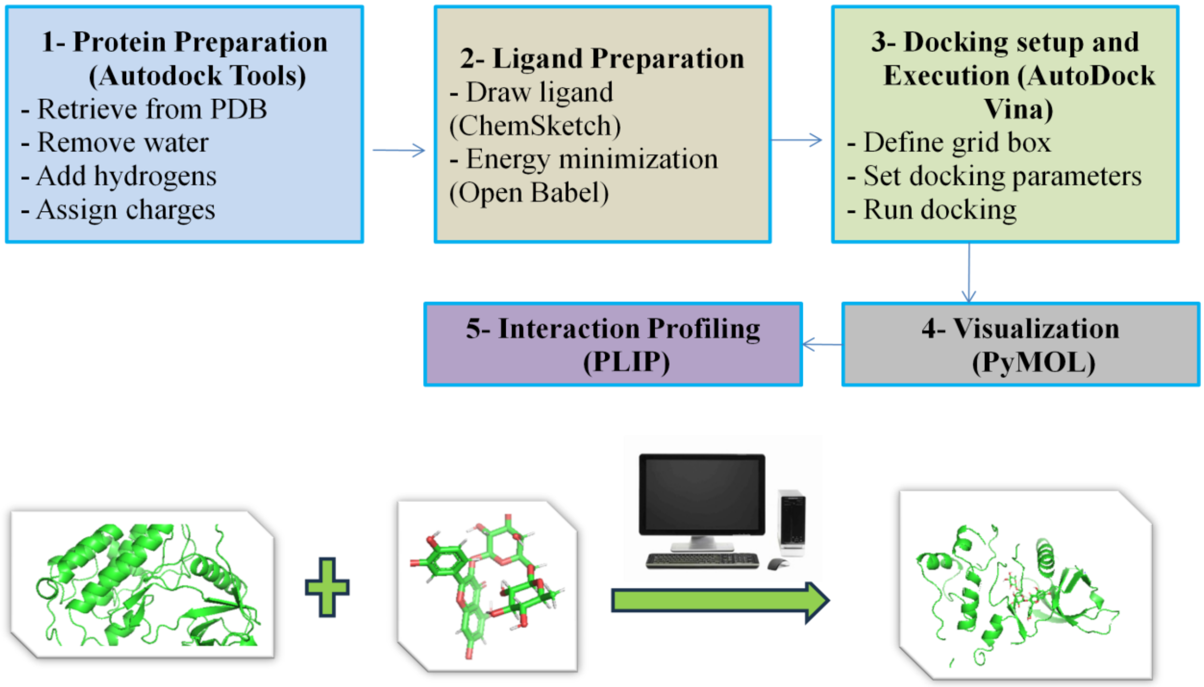

Figure S 13. Overview of the molecular docking workflow used in this study

The fragmentation of the identified compounds belonging to different classes of secondary metabolites

Benzimidazoles

The ESI-MS analysis revealed the presence of a molecular ion peak at *m/z* 192 $[M+H]^+$. The MS^2 spectrum showed an abundant fragment ion at *m/z* 160 $[M+H-CH_3OH]^+$ and also displayed a characteristic radical ion at *m/z* 132 $[M+H-CH_3OCO]^+$. Therefore, compound (**1**) was tentatively identified as carbendazim [1, 2].

Flavonoids

Compound (**2**) was tentatively identified as quercetin glucoside as the ESI-MS spectrum displayed a molecular ion peak at *m/z* 463 in the negative ion mode. Furthermore, the MS^2 spectrum displayed an abundant radical aglycone ion $[Y_0-H]^{-\cdot}$ at *m/z* 300 produced from the precursor ion *m/z* 463. Moreover, the positive ion mode revealed a molecular ion peak at *m/z* 465 $[M+H]^+$. The fragmentation process revealed a prominent base peak at *m/z* 303 $[M+H-162]^+$ owing to the neutral loss of a hexose moiety. Also, fragments at *m/z* 184 ($C_8H_8O_5$) and 123 ($C_7H_7O_2$) were observed due to a previously reported retrocyclization pathway of flavonol giving rise to $[^{1,2}A]^+$ and $[^{1,2}B]^+$ fragments, respectively [3, 4]. Furthermore, compound (**23**) was identified as quercetin–hydroxy-methyl glutaryl hexoside and its isomer as they showed a molecular ion peak at *m/z* 609 $[M+H]^+$. The MS^2 spectrum showed fragment ion at *m/z* 303 corresponding to the aglycone quercetin owing to the loss of the hexose moiety with the hydroxy-methyl glutaryl residue. Another fragment appeared at *m/z* 464 $[M+H-144]^+$ owing to the neutral loss of the hydroxy-methyl glutaryl substituent [5].

Acetophenones

The ESI-MS spectrum revealed two quasimolecular ion peaks corresponding to *m/z* 197 $[M+H]^+$ and *m/z* 219 $[M+Na]^+$. The MS^2 spectrum presented a radical ion at *m/z* 179 $[M+H-OH_2]^+$ corresponding to the loss of a water molecule (18 amu). Also, a radical ion was observed at *m/z* 137 $[M+H-OH-CH_3CO]^+$. Moreover, a radical ion was observed at *m/z* 107 corresponding to $(C_7H_7O)^{\cdot}$. Thus, compound (**3**) was tentatively identified as dimethoxy hydroxyacetophenone [6].

Phenolic acids

Compound (**4**) was tentatively identified as dicaffeoyl quinic acid as the ESI-MS spectrum showed a molecular ion peak at *m/z* 515 $[M-H]^-$ and a fragement ion was observed at *m/z* 353 $[M-H-162]^-$ due to the neutral loss of a caffeoyl moiety [7]. Furthermore, compound (**5**) was tentatively identified as ethyl caffeate. The ESI-MS spectrum displayed a molecular ion

peaks at *m/z* 209 $[M+H]^+$ and 207 $[M-H]^-$. The MS^2 spectrum of the precursor ion at *m/z* 207 showed several fragment ions. A radical ion was observed at *m/z* 179 $[M-H-C_2H_5]^{-\cdot}$, another radical ion was also observed at *m/z* 135 due to the subsequent loss of 44 amu corresponding to CO_2. Moreover, a characteristic peak was observed at *m/z* 161 corresponding to [M-H-ethanol]$^{-\cdot}$ [8, 9]. Moreover, compound (**8**) tentatively identified as ferulic acid pentosyl exhibited a molecular ion peak at *m/z* 325 $[M-H]^-$. A product ion was observed at *m/z* 193 corresponding to neutral loss of pentosyl moiety [10, 11]. Compound (**14**) was tentatively identified as tri-caffeoyl-anhydro-octulopyranosonic acid. The compound was identified by the appearance of a molecular ion peak at *m/z* 721 $[M-H]^-$. The MS^2 spectrum displayed a fragment ion at *m/z* 397 corresponding to the loss of two caffeoyl moieties. Subsequent fragmentation gives rise to radical ions at *m/z* 277 and 119 corresponding to ($C_{10}H_{13}O_9$) and (C_8H_7O), respectively [12].

Triterpenes and steroids

Compound (**10**) showed a molecular ion peak *m/z* 517 $[M-H]^-$. The MS^2 spectrum revealed fragment ions at *m/z* 455, 437 and 429 corresponding to $[M-H-CO_2-H_2O]^-$, $[M-H-CO_2-H_2O-H_2O]^-$ and $[M-H-2CO_2]^-$, respectively. Thus, compound (**10**) was tentatively identified as zahnic acid [13].

Chlorophyll catabolites

Compound (**18**) was tentatively identified as pheophorbide B. The ESI-MS spectrum showed a molecular ion peak at *m/z* 607 $[M+H]^+$ and a characteristic prominent product ion at *m/z* 547 $[M+H-CH_3COOH]^+$. In addition, The ESI-MS spectrum revealed a molecular ion peak at *m/z* 593 $[M+H]^+$ and showed a prominent characteristic fragment ion at *m/z* 533 $[M+H-CH_3COOH]^+$. Thus, compound (**24**) was identified as pheophorbide A [14].

Secoiridoids

Compound (**17**) displayed a quasimolecular ion peak at *m/z* 579 $[M+Na]^+$. Also, a molecular ion was observed at *m/z* 555 $[M-H]^-$. Additionally, the MS^2 spectrum showed fragment ions at *m/z* 225, 153 and 135. Therefore, the compound was identified as hydroxyoleuropein [15].

Fatty acids and their derivatives

Compound (**6**) was tentatively identified as corchorifatty acid F (trihydroxy octadecadienoic acid). The positive ion mode showed a quasimolecular ion peak at *m/z* 351 $[M+Na]^+$. Moreover, the negative ion mode showed a molecular ion at *m/z* 327 $[M-H]^-$. Fragmentation of the precursor ion at *m/z* 327 produced a fragment ion at *m/z* 291 $[M-H-2\ H_2O]^-$ owing to

the loss of two water molecules. Also, a product ion was displayed at *m/z* 229 ($C_{12}H_{21}O_4$) attributed to the allyl scission and *β*- fission of a hydroxyl group. Fragment ions were also observed at *m/z* 211 ($C_{12}H_{19}O_3$) and 171 ($C_9H_{15}O_3$) [16-18]. In addition, compound (**7**) was tentatively identified as trihydroxyoctadecenoic acid. A pseudomolecular ion peak was observed at *m/z* 353 $[M+ Na]^+$ in the positive ion mode and another one at *m/z* 329 in the negative ion mode. The MS^2 spectrum revealed a product ion attributed to the loss of HO-CH=CH$(CH_2)_3CH_3$ moiety corresponding to 100 amu at *m/z* 229 in the negative ion mode. Subsequent loss of $CH_2CH(OH)CH_2$ group corresponding to 58 amu gives rise to a product ion at *m/z* 171[18, 19]. Moreover, compound (**9**) was tentatively identified as octadecadienoic acid ethyl ester. The ESI-MS spectrum displayed a molecular ion peak at *m/z* 307 $[M\text{-}H]^-$. The MS^2 spectrum revealed a radical ion at *m/z* 235 $[M\text{-}H\text{-} C_2H_5OCO]^{-\cdot}$. Subsequent loss of C_5H_{11} give rise to a radical ion at *m/z* 71. Also fragments at *m/z* 185 and 125 were observed corresponding to ($C_{11}H_{21}O_2$) and (C_9H_{17}), respectively [20].

Additionally, compounds (**11**) and (**12**) were tentatively identified as hydroxy eicosapentaenoic acid and its isomer. A pseudomolecular ion was displayed at *m/z* 319 $[M+H]^+$. A product ion was revealed at *m/z* 274 $[M+H\text{-}COOH]^+$. Moreover another fragment ion was observed at *m/z* 230 $[M+H\text{-}C_4H_9O_2]^+$. Also, fragment ions were observed at *m/z* 85 and 57 [21]. Furthermore, compound (**13**) was tentatively identified as nonatriacontanoic acid. A molecular ion peak was observed at *m/z* 577.5 $[M\text{-}H]^-$. The MS^2 spectrum showed a radical ion at *m/z* 71 corresponding to (C_5H_{11}). Also, fragment ions were observed at *m/z* 95 and 81 [22].

Compounds (**15**) and (**16**) which appeared at retention times 14.05 and 14.17 min. were tentatively identified as hydroxy octadecatrienoic acid and its isomer. The compounds displayed a pseudomolecular ion at *m/z* 317 $[M+Na]^+$. Also, a molecular ion peak appeared in the negative ion mode at *m/z* 293. MS^2 spectrum of the negative ion mode showed a fragment ion at *m/z* 275 $[M\text{-}H\text{-}OH_2]^-$. Also, radical ions were observed at *m/z* 121 and 97 corresponding to (C_9H_{11}) and (C_7H_{13}), respectively, due to the observed cleavage at the conjugated triene [23]. Furthermore, compound (**20**) was tentatively identified as hydroxy palmitic acid. The ESI-MS spectrum revealed a molecular ion peak at *m/z* 271 $[M\text{-}H]^-$ and displayed fragment ion at *m/z* 225 [24].

Fatty amides

Compound (**19**) was tentatively identified as linoleamide. The ESI-MS spectrum showed a molecular ion peak at *m/z* 280 $[M+H]^+$. The MS^2 spectrum displayed fragment ions at *m/z* 263 and 245 corresponding to $[M+H-NH_3]^+$ and $[M+H-NH_3-H_2O]^+$, respectively. Also, a fragment ion is observed at *m/z* 95 [25]. Moreover, compound (**21**) showed a molecular ion peak at *m/z* 256 $[M+H]^+$. The MS^2 spectrum displayed fragment ions at *m/z* 102 and 88 corresponding to $[M+H-C_{11}H_{22}]^+$ and $[M+H-C_{11}H_{22}-CH_2]^+$, respectively. Also, a fragment ion was observed at *m/z* 43 corresponding to (C_3H_7). Thus, compound (**21**) was tentatively identified as palmitamide [26]. Furthermore, compound (**22)** was tentatively identified as oleamide. A molecular ion peak was observed at *m/z* 282 $[M+H]^+$. The MS^2 spectrum displayed fragment ions at *m/z* 265 and 247 corresponding to $[M+H-NH_3]^+$ and $[M+H-NH_3-H_2O]^+$, respectively [26]. Additionally, compound (**25**) displayed a pseudomolecular ion $[M+H]^+$ at *m/z* 284. The MS^2 spectrum revealed fragment ions at *m/z* 130, 116, 102 and 71. Thus, the compound was tentatively identified as octadecanamide (stearamide) [27].

References

1. Blasco, C., Fernández, M., Picó, Y., Font, G. & Mañes, J. Simultaneous determination of imidacloprid, carbendazim, methiocarb and hexythiazox in peaches and nectarines by liquid chromatography–mass spectrometry. *Anal. Chim. Acta* **461**, 109-116 (2002).

2. Grujic, S., Radisic, M., Vasiljevic, T. & Lausevic, M.. Determination of carbendazim residues in fruit juices by liquid chromatography-tandem mass spectrometry. *Food Addit. Contam.* **22**, 1132-1137 (2005).

3. Fabre, N., Rustan, I., de Hoffmann, E. & Quetin-Leclercq, J. Determination of flavone, flavonol, and flavanone aglycones by negative ion liquid chromatography electrospray ion trap mass spectrometry. *J. Am. Soc. Mass Spectrom.* **12**, 707-715 (2001).

4. Santos, S. A., Freire, C. S., Domingues, M., Silvestre, A. & Neto, C. Characterization of phenolic components in polar extracts of *Eucalyptus globulus* Labill. bark by high-performance liquid chromatography–mass spectrometry. *J. Agric. Food Chem.* **59**, 9386-9393 (2011).

5. Barreca, D., Gattuso, G., Laganà, G., Leuzzi, U. & Bellocco, E. *C*- and *O*-glycosyl flavonoids in Sanguinello and Tarocco blood orange (*Citrus sinensis* (L.) Osbeck) juice: Identification and influence on antioxidant properties and acetylcholinesterase activity. *Food Chem.* **196**, 619-627 (2016).

6. Szatmári, Á. *et al.* A pattern-triggered immunity-related phenolic, acetosyringone, boosts rapid inhibition of a diverse set of plant pathogenic bacteria. *BMC Plant Biol.* **21**, 1-20 (2021).

7. Carlotto, J. *et al.* Identification of a dicaffeoylquinic acid isomer from *Arctium lappa* with a potent anti-ulcer activity. Talanta **135**, 50-57 (2015).

8. Barth, C. d. *et al.* RP-HPLC and LC–MS–MS determination of a bioactive artefact from *Ipomoea pes-caprae* extract. Rev. Bras. Farmacogn. **29**, 570-577 (2019).

9. Wu, Z. *et al.* Analysis of caffeic acid derivatives from *Osmanthus yunnanensis* using electrospray ionization quadrupole time-of-flight mass spectrometry. *Eur. J. Mass Spectrom.* **15**, 415-429 (2009).

10. Aouey, B., Samet, A. M., Fetoui, H., Simmonds, M. S. & Bouaziz, M.. Anti-oxidant, anti-inflammatory, analgesic and antipyretic activities of grapevine leaf extract (*Vitis vinifera*) in mice and identification of its active constituents by LC–MS/MS analysis. *Biomed. pharmacother.* **84**, 1088-1098 (2016).

11. Verardo, V., Bonoli, M., Marconi, E. & Caboni, M.. Distribution of Bound Hydroxycinnamic Acids and Their Glycosyl Esters in Barley (*Hordeum vulgare* L.) Air-Classified Flour: Comparative Study between Reversed Phase-High Performance Chromatography - Mass Spectrometry (RP-HPLC/MS) and Spectrophotometric Analysis. *J. Agric. Food Chem.* **56**, 11900-11905 (2008).

12. Liao, S. *et al.* Rapid screening and identification of caffeic acid and its esters in *Erigeron breviscapus* by ultra-performance liquid chromatography/tandem mass spectrometry. *Rapid Commun Mass Spectrom.* **24**, 2533-2541 (2010).

13. Maisto, M. *et al.* Optimization of ursolic acid extraction in oil from *Annurca* apple to obtain oleolytes with potential cosmeceutical application. *Antioxid.* **12**, 224 (2023).

14. Vencl, F.V., Gómez, N.E., Ploss, K. & Boland, W. The chlorophyll catabolite, pheophorbide a, confers predation resistance in a larval tortoise beetle shield defense. *J. Chem. Ecol.* **35**, 281-288 (2009).

15. El-shazly, M. A., Hamed, A. A., Kabary, H. A., Ghareeb, M. A.. LC-MS/MS profiling, antibiofilm, antimicrobial and bacterial growth kinetic studies of *Pluchea dioscoridis* extracts. Acta Chromatogr. **3**, 338-350 (2021).

16. Yoshikawa, M. *et al.* Medicinal foodstuffs. XIV. On the bioactive constituents of moroheiya.(2): New fatty acids, corchorifatty acids A, B, C, D, E, and F, from the leaves of *Corchorus olitorius* L.(Tiliaceae): Structures and inhibitory effect on NO production in mouse peritoneal macrophages. *Chem. Pharm. Bull.* **46**, 1008-1014 (1998).

17. Yang, N., Yang, Y. & Li, K. Analysis of Hydroxy Fatty Acids from the Pollen of *Brassica campestris* L. var. oleifera DC. by UPLC-MS/MS. *J. Pharm.* 2013, 874875 (2013).

18. He, J., Dong, Y., Liu, X., Wan, Y., Gu, T., Zhou, X., Liu, M.. Comparison of chemical compositions, antioxidant, and anti-photoaging activities of *Paeonia suffruticosa* flowers at different flowering stages. *Antioxid.* **8**, 345 (2019).

19. Levandi, T., Püssa, T., Vaher, M., Toomik, P. & Kaljurand, M.. Oxidation products of free polyunsaturated fatty acids in wheat varieties. *Eur. J. Lip Sci. Technol.* **111**, 715-722 (2009).

20. Pichini, S. *et al.* Liquid chromatography–tandem mass spectrometry for fatty acid ethyl esters in meconium: Assessment of prenatal exposure to alcohol in two European cohorts. *J. Pharm. Biomed. Anal.* **48**, 927-933 (2008).

21. Masoodi, M., Mir, A. A., Petasis, N. A., Serhan, C. N. & Nicolaou, A. Simultaneous lipidomic analysis of three families of bioactive lipid mediators leukotrienes, resolvins, protectins and related hydroxy-fatty acids by liquid chromatography/electrospray ionisation tandem mass spectrometry. *Rapid Commun Mass Spectrom.* **22**, 75-83 (2008).

22. Hamdan, D. *et al.* Chemical profiles with cardioprotective and anti-depressive effects of *Morus macroura* Miq. leaves and stem branches dichloromethane fractions on isoprenaline induced post-MI depression. *RSC adv.* **12**, 3476-3493 (2022).

23. Xia, C. *et al.* Comprehensive profiling of macamides and fatty acid derivatives in maca with different postharvest drying processes using UPLC-QTOF-MS. *ACS omega* **6**, 24484-24492 (2021).

24. Kokotou, M., Mantzourani, C., Bourboula A., Mountanea, O. & Kokotos, G. A liquid chromatography-high resolution mass spectrometry (LC-HRMS) method for the determination of free hydroxy fatty acids in cow and goat milk. *Molecules* **25**, 3947 (2020).

25. Bertin, M. J., Zimba, P. V., Beauchesne, K. R., Huncik, K. M. & Moeller, P. D. Identification of toxic fatty acid amides isolated from the harmful alga *Prymnesium parvum* carter. Harmful Algae **20**, 111-116 (2012).

26. Nichols, K. K., Ham, B. M., Nichols, J. J., Ziegler, C. & Green-Church, K. B. Identification of fatty acids and fatty acid amides in human meibomian gland secretions. *Invest. Ophthalmol Vis Sci.* **48**, 34-39 (2007).

27. Castillo-Peinado, L., López-Bascón, M. A., Mena-Bravo, A., de Castro, M. D. & Priego-Capote, F. Determination of primary fatty acid amides in different biological fluids by LC–MS/MS in MRM mode with synthetic deuterated standards: influence of biofluid matrix on sample preparation. *Talanta* **193**, 29-36 (2019).
